# Platinum‐Templated Coupling of B=N Units: Synthesis of BNBN Analogues of 1,3‐Dienes and a Butatriene

**DOI:** 10.1002/anie.202106161

**Published:** 2021-06-24

**Authors:** Carina Brunecker, Merle Arrowsmith, Felipe Fantuzzi, Holger Braunschweig

**Affiliations:** ^1^ Institute for Inorganic Chemistry Julius-Maximilians-Universität Würzburg Am Hubland 97074 Würzburg Germany; ^2^ Institute for Sustainable Chemistry & Catalysis with Boron Julius-Maximilians-Universität Würzburg Am Hubland 97074 Würzburg Germany

**Keywords:** 1,3,2,4-diazadiboretidin-2-yl ligand, A-frame complex, B−N coupling, butatriene analogue, isosterism

## Abstract

The 1:2 reaction of [μ‐(dmpm)Pt(nbe)]_2_ (dmpm=bis(dimethylphosphino)methane, nbe=norbornene) with Cl_2_BNR(SiMe_3_) (R=tBu, SiMe_3_) yields unsymmetrical (N‐aminoboryl)aminoboryl Pt^I^_2_ complexes by B−N coupling via ClSiMe_3_ elimination. A subsequent intramolecular ClSiMe_3_ elimination from the tBu‐derivative leads to cyclization of the BNBN unit, forming a unique 1,3,2,4‐diazadiboretidin‐2‐yl ligand. In contrast, the analogous reaction with Br_2_BN(SiMe_3_)_2_ leads, via a twofold BrSiMe_3_ elimination, to a Pt^II^_2_ A‐frame complex bridged by a linear BNBN isostere of butatriene. Structural and computational data confirm π electron delocalization over the entire BNBN unit.

## Graphical abstract

The [μ‐(dmpm)Pt]_2_ template promotes the coupling of B=N units derived from dihalo(silylamino)borane precursors by B−N bond formation through intermolecular halosilane elimination. For Cl_2_BNR(SiMe_3_) η^1^‐(*N*‐aminoboryl)aminoboryl and η^1^‐1,3,2,4‐diazaboretidin‐2‐yl Pt^I^_2_ complexes are obtained, whereas for Br_2_BN(SiMe_3_)_2_ an A‐frame complex bridged by the first BNBN analogue of a butatriene is formed, as confirmed by structural and theoretical analyses.

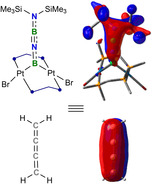

The replacement of C=C double bonds in organic molecules by isosteric covalent B=N units is not only interesting from a fundamental point of view, but also opens up the exploration of a vast hybrid organic–inorganic chemical space. While the typical B=N double bond (1.39 Å)^[1]^ is only marginally longer than a C=C double bond (1.34 Å, Figure 1), the intrinsic strong polarization of B−N bonds imparts very different electronic properties and stability to the resulting molecules and materials, which can be exploited for new applications in materials science, catalysis, and medicinal chemistry.

**Figure 1:**
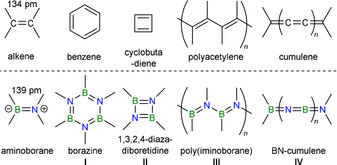
Conjugated organic systems and their all‐BN isosteres.

Since the landmark synthesis of borazine by Stock and Pohland in 1926 (Figure 1, **I**),^[2]^ new synthetic methodologies have enabled access to an ever‐increasing variety of B=N/ C=C‐isosteric compounds and materials, including boron nitride^[3]^ and borocarbonitride (B_*x*_C_*y*_N_*z*_) nanomaterials,^[4]^ hybrid organic–inorganic BN‐doped conjugated polymers,^[5]^ (poly)aromatic compounds,^[6]^ and aromatic small molecules.^[7]^ However, well‐defined acyclic conjugated BN chains, such as poly(iminoboranes) (**III**) or BN‐based cumulenes (**IV**), remain difficult to access. The intuitive synthetic routes to **III** via the polymerization of iminoborane (RB≡NR′) precursors^[8]^ or the dehydrocoupling of amine borane (H_2_RB⋅NH_2_R′) precursors^[9]^ are in practice marred by the formation of cyclic oligomers such as **I** and **II**. The most efficient access to higher oligo(iminoboranes) is by B−N coupling of chloroborane and silylamine precursors via ClSiMe_3_ elimination.^[10]^ The group of Helten has used this methodology to synthesize the first well‐defined oligo(iminoboranes) (**V**) by polycondensation of 1,3‐bis(trimethylsilyl)‐1,3,2‐diazaborolidine precursors with dichloro(organo)boranes (Scheme 1 a).^[11]^ Our group has also reported the coupling of two Cl_2_BN(SiMe_3_)_2_ molecules at [(C_5_H_5_)Ru(CO)_2_]Na with elimination of NaCl and ClSiMe_3_, yielding the (*N*‐aminoboryl)aminoboryl complex **VI** (Scheme 1 b).^[12]^

**Scheme 1:**
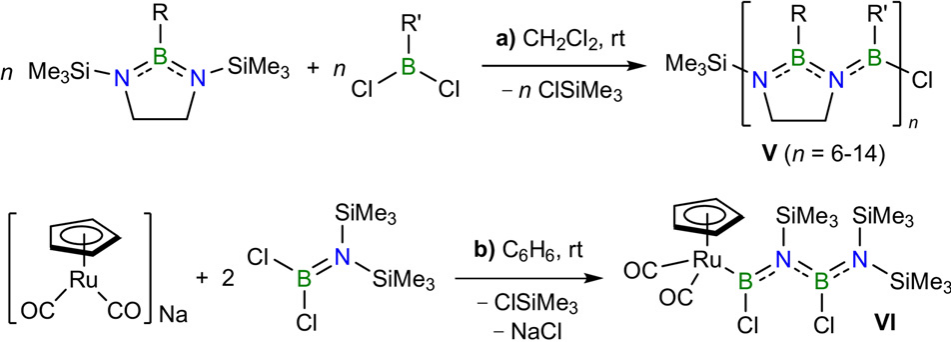
Examples of syntheses of oligo(iminoboranes) by B−N coupling via ClSiMe_3_ elimination.

We have recently reported the synthesis of the boranediyl A‐frame complexes **2‐X^Y^** from the twofold oxidative addition of dihaloborane precursors (X_2_BY, X=Cl, Br, I; Y=X, alkyl, aryl, amino) to the bis(dimethylphosphino)methane (dmpm)‐bridged Pt^0^_2_ complex **1** (Scheme 2 a).^[13]^ Inspired also by the metal‐templated coupling of two BN units at ruthenium in complex **VI** (Scheme 1 b),^[12]^ we now report the use of the Pt_2_(dmpm)_2_ scaffold as a template for the coupling of B=N units derived from the coupling of dihalo(silylamino)boranes (X_2_BNR(SiMe_3_), X=Cl, Br; R=*t*Bu, SiMe_3_) by elimination of XSiMe_3_, ultimately leading to the isolation of the first BNBN‐cumulene, isosteric with butatriene.

**Scheme 2:**
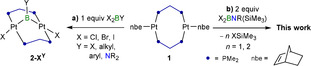
Synthesis of boranediyl‐bridged diplatinum A‐frame complexes.

Whereas the reaction of complex **1** with Cl_2_BNMe_2_ yields the aminoboranediyl‐bridged A‐frame complex **2‐Cl^NMe2^** (Scheme 2 a), the reactions of **1** with Cl_2_BNR(SiMe_3_) (R=*t*Bu, SiMe_3_) always proceeded in a 1:2 ratio. The resulting products **3** ^***t*****Bu**^ and **3^SiMe3^**, which precipitated as pale yellow solids, both display two broad ^11^B NMR resonances, at 53 (fwmh≈1280 Hz, Pt*B*) and 32 ppm (fwmh≈880 Hz, N_2_ *B*Cl) for **3** ^***t*****Bu**^, and 57 (fwmh≈1990 Hz, Pt*B*) and 33 ppm (fwmh≈750 Hz, N_2_ *B*Cl) for **3^SiMe3^** (Scheme 3 a). Complexes **3^R^** are reminiscent of complex **VI** (Scheme 1 b), which shows similar ^11^B NMR resonances at 60.3 and 35.0 ppm.^[12]^ The ^31^P{^1^H} spectra of **3^R^** show two multiplets with higher‐order satellites in a 1:1 ratio, at −14.3 (^1^ *J*_P‐Pt_ *=*3195 Hz, *P_2_* PtCl) and −29.9 ppm (^1^ *J*_P‐Pt_ *=*2733 Hz, *P_2_* PtB) for **3** ^***t*****Bu**^, and −14.3 (^1^ *J*_P‐Pt_ *=*3150 Hz, *P_2_* PtCl) and −29.6 ppm (^1^ *J*_P‐Pt_ *=*2708 Hz, *P_2_* PtB) for **3^SiMe3^**. X‐ray crystallographic analyses of single crystals of **3** ^***t*****Bu**^ confirmed the coupling of the two BN units at one platinum center (Figure 2). Due to systematic rotational disorder of the terminal B(Cl)N*t*Bu(SiMe_3_) moiety, structural parameters cannot be fully discussed. The Pt−Pt distance of 2.7067(6) Å, however, is clearly indicative of Pt−Pt bonding. The Pt2−B1 bond length of 2.039(6) Å is within the typical range for square planar platinum amino(chloro)boryl complexes (2.00–2.85 Å), while the B1−N1 bond of 1.421(8) Å is slightly longer than in these complexes (ca. 1.39 Å)^[14]^ due to the additional π electron delocalization over the entire BNBN unit in **3** ^***t*****Bu**^.

**Figure 2:**
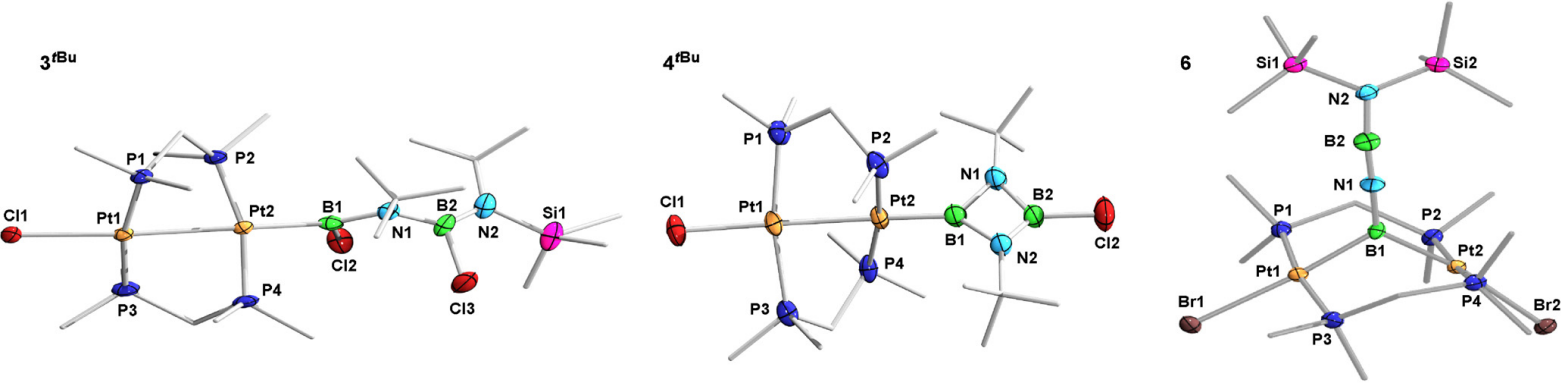
Crystallographically derived molecular structures of (from left to right) **3** ^***t*****Bu**^ (least disordered one of the two molecules of **3** ^***t*****Bu**^ in the asymmetric unit), **4** ^***t*****Bu**^, and **6**.^[26]^ Thermal ellipsoids at 50 % probability. Thermal ellipsoids of ligand periphery and hydrogen atoms omitted for clarity. Only the major part of the disorders in **3** ^***t*****Bu**^ (terminal B(Cl)N*t*Bu(SiMe_3_) moiety) and **4** ^***t*****Bu**^ (entire (BN*t*Bu)_2_Cl moiety and one dmpm ligand) is shown. Due to the restraints applied to these disorders during refinement, the structural parameters of **3** ^***t*****Bu**^ and **4** ^***t*****Bu**^ may not be fully discussed. Selected bond lengths (Å) and angles (°) for **3** ^***t*****Bu**^: Cl1–Pt1 2.4939(13), Pt1–Pt2 2.7067(6), Pt–P 2.2446(14)–2.2651(14), Pt2–B1 2.039(6), B1–N1 1.421(8), Cl1‐Pt1‐Pt2 172.32(3), P1‐Pt2‐B1 174.14(16), Σ(∡B1) 360.0(4), torsion angles P1‐Pt1‐Pt2‐P2 −47.8(4), P3‐Pt1‐Pt2‐P4 −54.32(5); for **4** ^***t*****Bu**^: Cl1–Pt1 2.535(3), Pt1–Pt2 2.7214(7); for **6**: Pt1⋅⋅⋅Pt2 3.2397(3), Pt1–B1 2.028(6), Pt2–B1 2.021(6), Pt1–Br1 2.6098(6), Pt2–Br2 2.6363(6), Pt–P 2.2679(15)–2.2913(14), B1–N1 1.396(7), N1–B2 1.237(8), B2–N2 1.388(8), Pt1‐B1‐Pt2 106.3(3), B1‐N1‐B2 173.8(6), N1‐B2‐N2 171.3(7), torsion angles P1‐Pt1‐Pt2‐P2 −12.29(5), P3‐Pt1‐Pt2‐P4 −23.83(5).

**Scheme 3:**
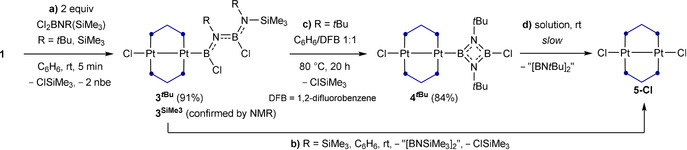
Reactions of complex **1** with Cl_2_BNR(SiMe_3_) (R=*t*Bu, SiMe_3_). Isolated yields in parentheses.

Complex **3^SiMe3^** could not be fully characterized as it decomposed rapidly in solution into ClSiMe_3_ and a number of dmpm‐containing platinum complexes, the known complex [μ‐(dmpm)PtCl]_2_ (**5‐Cl**: *δ*(^31^P)=−19.3 ppm, ^1^ *J*_P−Pt_=2650 Hz)^[13a]^ being the major decomposition product (Scheme 3 b, see Figure S18 in the SI). The fate of the remaining [BNSiMe_3_]_2_ fragment could not be determined as the ^11^B NMR spectrum of the final product mixture was silent, and a colorless by‐product, insoluble in all common organic solvents, was formed.^[15]^ In contrast, **3** ^***t*****Bu**^ was stable in solution at room temperature but selectively converted to **4** ^***t*****Bu**^ at 80 °C by intramolecular cyclization of the BNBN moiety under ClSiMe_3_ elimination (Scheme 3 c). This reaction is analogous to the cyclization of RClB−N(*t*Bu)−B(Cl)−N*t*Bu(SiMe_3_) (R=NMe_2_, NEt_2_, Et, *i*Bu) to 1,3,2,4‐diazadiboretidines by ClSiMe_3_ elimination, reported by Paetzold in 1988.^[16]^ The ^11^B NMR spectrum of **4** ^***t*****Bu**^ is nearly identical to that of **3** ^***t*****Bu**^, displaying two broad resonances at 54 (fwmh≈1480 Hz, Pt*B*) and 32 ppm (fwmh≈470 Hz, N_2_ *B*Cl). The conversion of **3** ^***t*****Bu**^ to **4** ^***t*****Bu**^ is evidenced more clearly by changes in the ^31^P{^1^H} spectrum, which shows two new 1:1 multiplets with higher‐order satellites, both shifted ca. 2 ppm downfield from **3** ^***t*****Bu**^, at −12.8 (^1^ *J*_P−Pt_ *=*3198 Hz, *P_2_* PtCl) and −27.6 ppm (^1^ *J*_P−Pt_ *=*2632 Hz, *P_2_* PtB), the ^1^ *J*_P−Pt_ coupling constant of the latter being ca. 100 Hz smaller than in **3** ^***t*****Bu**^. Crystallization attempts of **4** ^***t*****Bu**^ always yielded pseudo‐merohedrally twinned crystals (see solid‐state structure in Figure 2), in which the BNBN heterocycle presents a twofold disorder by rotation of about the Pt2−B1 bond, thus precluding any discussion of bond lengths and angles in this unit. Despite the well‐established chemistry of 1,3,2,4‐diazadiboretidines as η^4^‐ligands for transition metals,^[17]^ **3** ^***t*****Bu**^ represents a hitherto unknown binding mode of this type of ligand as an anionic η^1^‐ligand via coordination at boron. In solution at room temperature, compound **4** ^***t*****Bu**^ decomposed very slowly but selectively over a period of several weeks to complex **5‐Cl** and an unidentified intractable colorless solid, by formal loss of “[BN(*t*Bu)]_2_” (Scheme 3 d).^[15]^

To our surprise the reaction of **1** with Br_2_BN(SiMe_3_)_2_ resulted instead in the formation of the A‐frame complex **6**, isolated as a yellow solid in 46 % yield (Scheme 4).^[18]^ The ^11^B NMR spectrum of **6** displays two broad resonances at ca. 57 (fwmh≈1510 Hz) and 26 ppm (fwmh≈690 Hz), the former being attributed to the platinum‐bound boron nucleus by analogy with the ^11^B NMR shift of the related dimethylaminoboranediyl‐bridged A‐frame complex **2‐Br^NMe2^** (*δ*(^11^B)=52 ppm),^[13]^ the latter to the dicoordinate N*B*N boron nucleus. The ^31^P{^1^H} NMR spectrum showed a singlet at −7.1 ppm, close to that of **2‐Br^NMe2^** (*δ*(^31^P)=−5.6 ppm), with a higher‐order satellite splitting pattern typical for A‐frame complexes (^1^ *J*_P−Pt_ *=*3568 Hz, ^3^ *J*_P−Pt_ *=*272 Hz, ^1^ *J*_Pt−Pt_ *=*1826 Hz). ^11^B and ^31^P{^1^H} NMR‐spectroscopic monitoring of the reaction showed no sign of formation of the bromide analogue of **3^SiMe3^**.

**Scheme 4:**
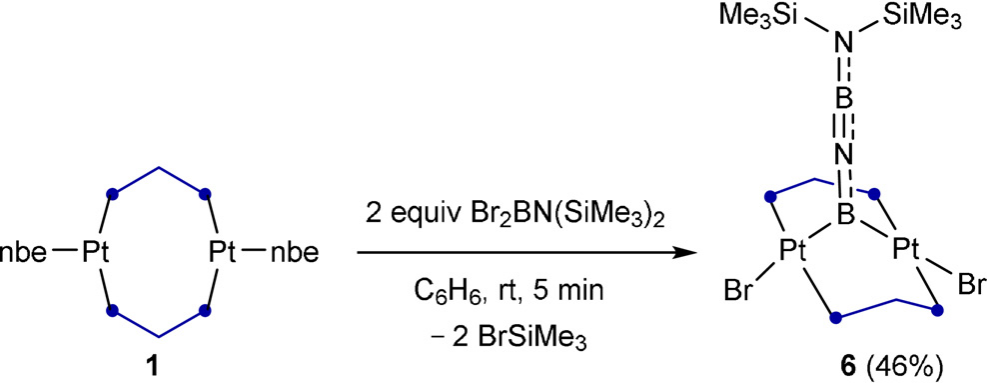
Reaction of complex **1** with BBr_2_N(SiMe_3_)_2_. Isolated yield in parentheses.

We propose that the formation of complexes **3^R^** and **6** proceeds via a same intermediate η^1^‐(silylamino)haloboryl complex **Int‐X^R^** formed by the oxidative addition of X_2_BNR(SiMe_3_) to **1** (Scheme 5).^[19]^ This step can be followed either by B−N coupling with a second equivalent X_2_BNR(SiMe_3_) via XSiMe_3_ elimination (reaction rate constant *k*_a_) to form an η^1^‐(*N*‐aminoboryl)aminoboryl complex analogous to **3^R^**, or by the oxidative addition of the second B−X bond of the silylamino(halo)boryl ligand to platinum to form the (silylamino)boranediyl A‐frame complex **2‐X^NR(SiMe3)^** (reaction rate constant *k*_b_). For R=SiMe_3_, the latter then undergoes twofold XSiMe_3_ elimination with a second equivalent of X_2_BN(SiMe_3_)_2_ to form complex **6**. The selectivity of the reaction is therefore determined by the relative values of the reaction rate constants *k*_a_ and *k*_b_: for X=Cl the rate of B−N coupling outperforms that of oxidative addition of B−Cl to Pt, leading to the exclusive formation of **3^R^**, the opposite being the case for X=Br, leading to the exclusive formation of **6**.

**Scheme 5:**
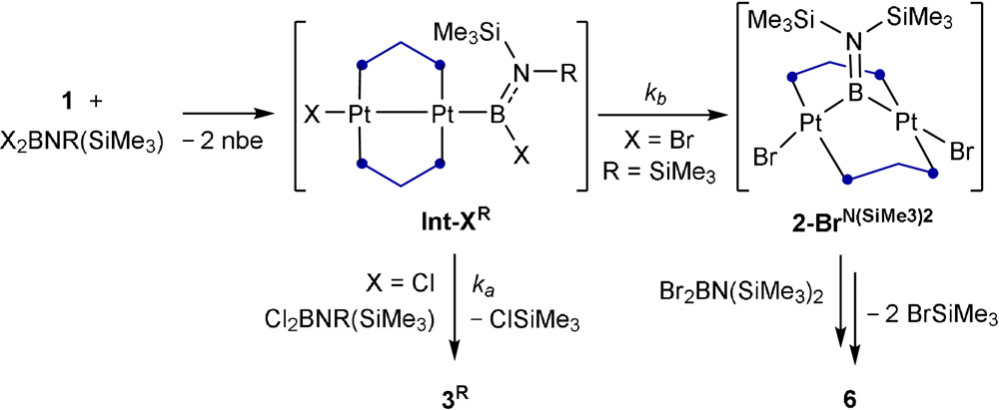
Proposed mechanism of formation of **3^R^** and **6** via the common intermediate **Int‐X^R^**.

The solid‐state structure of **6** (Figure 2) confirmed the formation of the near‐linear BNBN unit bridging the two platinum centers (B1‐N1‐B2 173.8(6), N1‐B2‐N2 171.3(7)°). While the Pt−B bond lengths of 2.028(6) and 2.021(6) Å are similar to those in complex **2‐Br^NMe2^** (2.028(10), 2.042(9) Å), the A‐frame structure itself is more strongly distorted from the ideal A‐frame than in **2‐Br^NMe2^**, as evident in the much shorter Pt⋅⋅⋅Pt distance (**6** 3.2397(3); **2‐Br^NMe2^** 3.3003(4) Å) and larger P1/3‐Pt1‐Pt2‐P2/4 torsion angles (**6** −12.29(5), −23.83(5)°; **2‐Br^NMe2^** 4.96(7), 15.62(8)°).^[13]^ Furthermore, the B1−N1 and B2−N2 bond lengths of 1.396(7) and 1.388(8) Å are within the range of partial double bonds, whereas the central N1−B2 bond is significantly shorter (1.237(8) Å), corresponding to a partial triple bond.^[1]^ While the linear BNBN motif can be viewed formally as a 1‐boryl‐2‐(amino)iminoborane, the delocalization of the π electron density apparent in the B−N bond lengths makes it structurally more akin to an all‐BN isostere of a butatriene. Unlike butatriene, however, which is fully planar, the B1 and N2 planes form an angle of ca. 24°, which could result from the steric repulsion between the SiMe_3_ groups and the dmpm ligands.

The electronic structure of **6** was further investigated using DFT and intrinsic bond orbital (IBO)^[20]^ calculations. The BNBN motif in the optimized structure of **6**, obtained at the M06^[21]^‐D3^[22]^/cc‐pVDZ^[23]^,aug‐cc‐pVDZ‐PP{Pt}^[24]^ level of theory, shows a larger deviation from linearity (B1‐N1‐B2 161.3°, N1‐B2‐N2 176.2°) than that of the solid‐state structure. Similar results were obtained with other density functionals (see details in the SI). In order to investigate the origin of this deviation, we performed computations on four truncated model systems, in which the PMe_2_ and SiMe_3_ groups were successively replaced with PH_2_ and SiH_3_ or H, respectively (see Figure S19 in the SI). In all of these cases, the BNBN moiety was found to be linear (B1‐N1‐B2 and N1‐B2‐N2 178.8–180.0°). The distortion from linearity therefore seems to arise from the steric repulsion between the PMe_2_ and SiMe_3_ substituents, although the additional influence of crystal packing forces in the solid‐state structure cannot be discounted. Furthermore, the calculated Mayer bond orders (MBOs)^[25]^ of the BNBN motif in **4** (B1−N1: 1.38, N1−B2: 2.11, B2−N2: 1.32) are very similar to those obtained for the parent H_2_BNBNH_2_ system (B1−N1: 1.51, N1−B2: 2.13, B2−N2: 1.43), these values suggesting strong cumulenic character in both cases. Indeed, inspection of the IBOs of **6** (Figure 3 a) reveals that IBO‐1 and IBO‐3, which are orthogonal to the (Pt1‐B1‐Pt2) plane, are partially delocalized to the neighboring B2 and B1 atoms, evidencing deviation from the 1‐boryl‐2‐(amino)iminoborane picture. This view is also supported by inspection of the canonical Kohn–Sham molecular orbitals (MOs) of **6** and H_2_BNBNH_2_ (Figure 3 b and S20 in the SI), where π electron delocalization over the entire BNBN unit is observed. The description of **6** as a BNBN analogue of butatriene is, therefore, fully supported by quantum chemical investigations.

**Figure 3:**
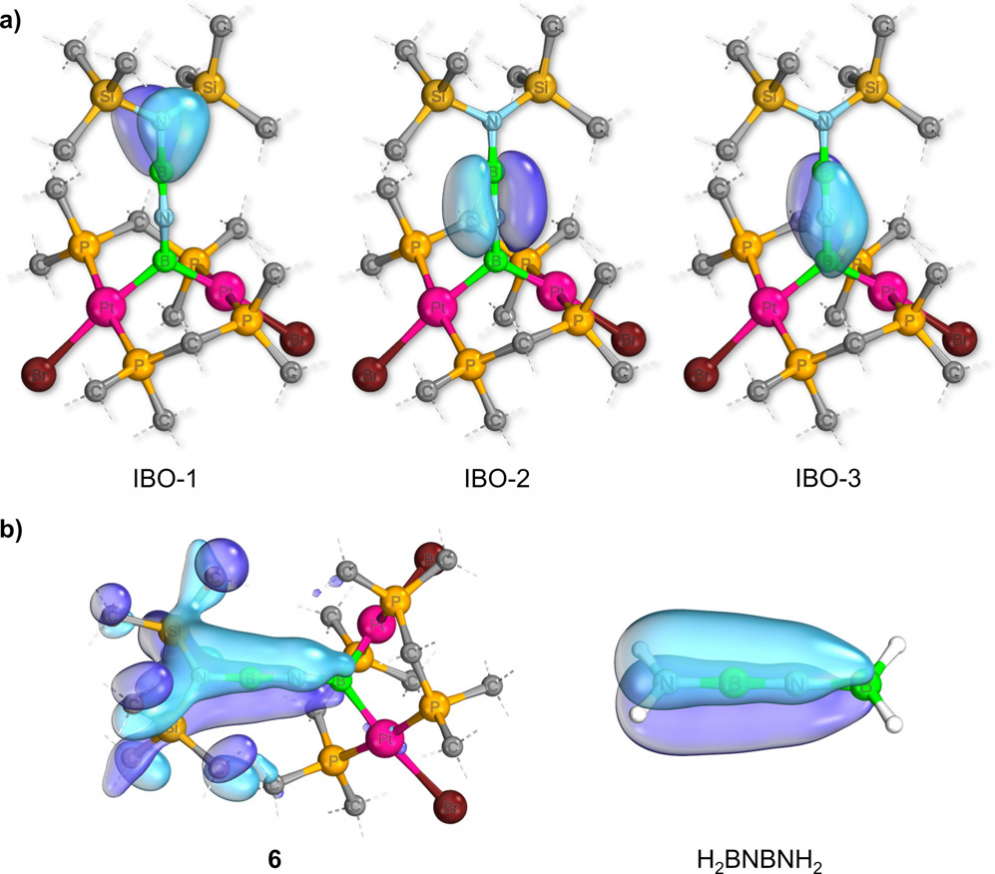
a) Selected IBOs of **6**. b) The fully π‐delocalized MOs of **6** (left, HOMO−30) and H_2_BNBNH_2_ (right, HOMO−3), highlighting the cumulenic character of their BNBN motifs.

To conclude, we have shown that the [μ‐(dmpm)Pt]_2_ framework acts as an effective template for the coupling of B=N units obtained by the intermolecular B−N coupling of dihalo(silylamino)boranes via halosilane elimination. For Cl_2_BNR(SiMe_3_) precursors BN chain growth occurs at a side‐on Pt^I^_2_ complex, whereas for Br_2_BN(SiMe_3_)_2_ an A‐frame Pt^II^_2_ complex bridged by a linear BNBN unit is formed. Structural and computational analyses confirm a cumulenic motif isosteric with butatriene.

## Conflict of interest

The authors declare no conflict of interest.

## Supporting information

As a service to our authors and readers, this journal provides supporting information supplied by the authors. Such materials are peer reviewed and may be re‐organized for online delivery, but are not copy‐edited or typeset. Technical support issues arising from supporting information (other than missing files) should be addressed to the authors.

Supplementary
