# Supplementary material for: Platinum‐Templated Coupling of B=N Units: Synthesis of BNBN Analogues of 1,3‐Dienes and a Butatriene
Source: Angew Chem Int Ed Engl. 2021 Jun 24;60(31):16864–8. doi: 10.1002/anie.202106161 (PMC8362192; doi:10.1002/anie.202106161)
Supplement: Supplementary file 1 — Supplementary [file ANIE-60-16864-s001.pdf]

Angewandte Chemie
Eine Zeitschrift der Gesellschaft Deutscher Chemiker

Supporting Information

Platinum-Templated Coupling of B=N Units: Synthesis of BNBN Analogues of 1,3-Dienes and a Butatriene

*Carina Brunecker, Merle Arrowsmith, Felipe Fantuzzi, and Holger Braunschweig**

anie_202106161_sm_miscellaneous_information.pdf

Supporting Information

Table of contents

Methods and materials 2

Synthetic procedures 3

NMR spectra of new compounds 6

Decomposition of **3**^{SiMe3} 23

X-ray crystallographic data 24

Computational details 27

Cartesian coordinates 29

References 49

Methods and materials

All manipulations were performed either under an atmosphere of dry argon or *in vacuo* using standard Schlenk line or glovebox techniques. Deuterated solvents were dried over molecular sieves and degassed by three freeze-pump-thaw cycles prior to use. All other solvents were distilled and degassed from appropriate drying agents. Solvents (both deuterated and non-deuterated) were stored under argon over activated 4 Å molecular sieves. NMR spectra were acquired on a Bruker Avance 500 NMR spectrometer (^{1}H and $^{1}H\{^{31}P\}$: 500.1 MHz, $^{11}B\{^{1}H\}$: 160.5 MHz, $^{13}C\{^{1}H\}$: 125.8 MHz, $^{31}P\{^{1}H\}$: 202.5 MHz, ^{15}N: 50.7 MHz), Bruker Avance 400 NMR spectrometer ($^{11}B\{^{1}H\}$: 128.4 MHz, $^{31}P\{^{1}H\}$: 162.0 MHz). Chemical shifts (δ) are given in ppm and internally referenced to the carbon nuclei ($^{13}C\{^{1}H\}$) or residual protons (^{1}H) of the solvent. $^{11}B\{^{1}H\}$, $^{31}P\{^{1}H\}$ and ^{15}N spectra were referenced to [$BF_3{\cdot}OEt_2$], 85% H_3PO_4 and CH_3NO_2, respectively, as external standards. Reported ^{15}N shifts were derived from ^{15}N-^{1}H HMBC spectra. Microanalyses (C, H, N) were performed on an Elementar vario MICRO cube elemental analyzer.

Solvents and reagents were purchased from Sigma Aldrich or Alfa Aesar. Deuterated solvents were degassed with three freeze-pump-thaw cycles and stored over molecular sieves in J. Young-style ampoules or in a glovebox. [{(μ-dmpm)Pt(nbe)}$_2$] (**1**, nbe = norbornene),[1] $BBr_2BN(SiMe_3)_2$,[2] $BCl_2N(SiMe_3)_2$,[3] BCl_2N(*t*Bu)($SiMe_3$)[4] and BBr_2OSiMe_3[5] were synthesized using literature procedures or modifications thereof.

Synthetic procedures

Syntheses of diplatinum A-frame boranediyl complexes

Notes: The isolated A-frame boranediyl complexes all decomposed in solution or under reduced pressure. Isolated material had to be dried by evaporation of the washing solvent (pentane or hexane) at ambient pressure. As a result the elemental analyses and NMR spectra all contain residual solvent. In all cases attempts to detect the ^{195}Pt NMR signal failed, presumably due to strong broadening caused by coupling to ^{31}P as well as the quadrupolar ^{10}B and ^{11}B boron nuclei.

[(μ-dmpm)$_2$ClPt$_2${BClN(*t*Bu)BClN(*t*Bu)(SiMe$_3$)}], 3^{tBu}

In a vial one equivalent of **1** (40.0 mg, 47.0 µmol) was dissolved in benzene (0.7 mL) and two equivalents of BCl$_2$N(*t*Bu)(SiMe$_3$) (21.3 mg, 94.0 µmol) were added. The mixture was shaken causing the immediate precipitation of a yellow solid. After filtration the solid was recrystallized from benzene/pentane and **3^{tBu}** (43.0 mg, 42.8 µmol, 91%) was isolated as a yellow solid. ^{1}H NMR (500.1 MHz, C$_6$D$_6$): δ = 2.30 (m + satellites, $^3J_{H\text{-}Pt}$ = 16.5 Hz, 2H, P$_2$CH_2), 2.13 (two overlapping m + satellites, $^3J_{H\text{-}Pt}$ = 23, 14.5 Hz, 2H, P$_2$CH_2), 1.70 (dd + satellites, $^2J_{H\text{-}P}$ = 4.2 Hz, $^4J_{H\text{-}P}$ = 2.5 Hz, $^3J_{H\text{-}Pt}$ = 35 Hz, 3H, PCH_3), 1.58−1.65 (m + satellites, $^3J_{H\text{-}Pt}$ = 34 Hz, 3H, *P*CH_3), 1.61 (s, 9H, C(CH_3)$_3$), 1.53−1.56 (m + satellites, 3H, *P*CH_3), 1.52 (s, 9H, C(CH_3)$_3$), 1.49−1.52 (m + satellites, 6H, *P*CH_3), 1.46–1.49 (m + satellites, 3H, *P*CH_3), 1.45 + 1.43 (two app. t + satellites, $^2J_{H\text{-}P}$ = $^4J_{H\text{-}P}$ = 3.0 Hz, 3H each, *P*CH_3), 0.68 (s + satellites, $^2J_{H\text{-}Si}$ = 6 Hz, $^5J_{H\text{-}Pt}$ = 119 Hz, 9H, Si(CH_3)$_3$) ppm. ^{13}C{^{1}H} NMR (100.6 MHz, C$_6$D$_6$): δ = 56.7 (s, N*C*(CH$_3$)$_3$), 55.0 (s + satellites, $^3J_{C\text{-}Pt}$ = 15 Hz, N*C*(CH$_3$)$_3$), 34.2–44.3 (m, P$_2$*C*H$_2$), 34.4 (s, N*C*(CH$_3$)$_3$), 32.2 (s + satellites, $^4J_{C\text{-}Pt}$ = 12 Hz, PtBNC(*C*H$_3$)$_3$), 17.8–20.2 (m + satellites, P$_2$*C*H$_2$), 19.3−18.8 (m + satellites, P*C*H$_3$), 16.1−16.7 (m + satellites, P*C*H$_3$), 8.6 (Si(*C*H$_3$)$_3$) ppm. ^{11}B{^{1}H} NMR (160.5 MHz, C$_6$D$_6$): δ = 53 (br, fwmh ≈ 1280 Hz, Pt−*B*), 32 (br, fwmh ≈ 880 Hz, N$_2$*B*Cl) ppm. ^{15}N NMR (50.7 MHz, C$_6$D$_6$, 297 K): δ = −224.7 (s, 1N, B$_2$*N*tBu), −252.3 (s, 1N, *N*(*t*Bu)(TMS)) ppm. ^{31}P{^{1}H} NMR (162.0 MHz, C$_6$D$_6$): δ = −14.3 (m + satellites, $^1J_{P\text{-}Pt}$ = 3195 Hz, $^3J_{P\text{-}Pt}$ = 69 Hz, 2P, *P*$_2$PtCl), −29.9 (m + satellites, $^1J_{P\text{-}Pt}$ = 2733 Hz, $^3J_{P\text{-}Pt}$ = 71 Hz, 2P, *P*$_2$PtB) ppm. Elemental analysis (%) calculated for [(C$_{21}$H$_{55}$B$_2$Cl$_3$N$_2$P$_4$Pt$_2$Si)·(C$_5$H$_{12}$)$_{0.6}$] (M$_W$ = 1149.1): C 27.48, H 5.98, N 2.67; found: C 27.48, H 5.88, N 2.44.

[(μ-dmpm)$_2$ClPt$_2${BClN(SiMe$_3$)BClN(SiMe$_3$)$_2$}], 3^{SiMe3}

In a vial one equivalent of **1** (30.0 mg, 35.3 µmol) was dissolved in benzene (0.7 mL) and two equivalents of BCl$_2$N(SiMe$_3$)$_2$ in benzene (17.1 mg, 70.5 µmol) were added. The mixture was then shaken, causing the immediate precipitation of **3^{SiMe3}** as a yellow solid. ^{11}B{^{1}H} NMR (160.5 MHz, CD$_2$Cl$_2$): δ = 57 (br, fwmh ≈ 1990 Hz), 30 (br, fwmh ≈ 750 Hz) ppm. ^{31}P{^{1}H} NMR (162.2 MHz, C$_6$D$_6$): δ = −14.3 (m + satellites, $^1J_{P\text{-}Pt}$ = 3150 Hz, $^3J_{P\text{-}Pt}$ = 71 Hz, 2P, *P$_2$*PtCl), −29.6 (m + satellites, $^1J_{P\text{-}Pt}$ = 2708 Hz, $^3J_{P\text{-}Pt}$ = 71 Hz, 2P, *P$_2$*PtB) ppm. *Note: Due to rapid decomposition in solution at rt (see Figure S18), **3^{SiMe3}** could not be fully characterized. Unfortunately, the formation of **3^{SiMe3}** did not proceed at temperatures below rt. Furthermore, low temperature NMR-spectroscopic analysis of the isolated product was prevented by rapid crystallization at temperatures below rt. The ^{11}B and ^{31}P NMR spectra provided in Figures S1 and S2 were obtained from the crude product immediately after completion of the reaction. The major decomposition products of **3^{SiMe3}** identifiable by NMR spectroscopy were the dichlorodiplatinum complex [µ-(dmpm)PtCl]$_2$ (**5-Cl**, δ(^{31}P) = –19.3 ppm, $^1J_{P\text{–}Pt}$ = 2650 Hz)[1] and ClSiMe$_3$. The fate of the [BNSiMe$_3$]$_2$ fragment could not be determined as the ^{11}B NMR spectrum of the final product mixture was silent, and a colorless by-product insoluble in all common organic solvents was formed.*

[(μ-dmpm)$_2$ClPt$_2${η^1-BN(*t*Bu)BClN(*t*Bu)}], 4^{tBu}

In a vial one equivalent of **3^{tBu}** (40.0 mg, 39.8 µmol) was dissolved in benzene/1,2-difluorobenzene (1:1) (0.7 mL) and the mixture was heated for 20 h at 80 °C. The mixture was then shaken, causing the immediate precipitation of a yellow solid. After recrystallization from benzene/1,2-difluorobenzene/pentane, **4^{tBu}** (30.0 mg, 33.4 µmol, 84%) was isolated as a yellow solid. ^{1}H NMR (500.1 MHz, C$_6$D$_6$): δ = 2.15 + 2.17 (two overlapping t + satellites, $^2J_{H\text{-}P}$ = 6.8 Hz, $^3J_{H\text{-}Pt}$ = 41 Hz, 4H, P$_2$C*H$_2$*), 1.51 (t + satellites, $^1J_{H\text{-}P}$ = $^3J_{H\text{-}P}$ = 3.2 Hz, $^3J_{H\text{-}Pt}$ = 36.5 Hz, 12H, *P*C*H*$_3$), 1.45 (t + satellites, $^1J_{H\text{-}P}$ = $^3J_{H\text{-}P}$ = 3.0 Hz, $^3J_{H\text{-}Pt}$ = 22 Hz, 12H, PC*H*$_3$), 1.37 (s, 18H, C(C*H$_3$*)$_3$) ppm. ^{13}C{^{1}H} NMR (125.8 MHz, C$_6$D$_6$): δ = 49.2 (s, N*C*(CH$_3$)$_3$), 42.4−43.0 (m + satellites, P$_2$*C*H$_2$), 33.2 (s, C(*C*H$_3$)$_3$), 17.8–18.6 (m + satellites, P*C*H$_3$), 16.4−17.0 (m + satellites, P*C*H$_3$) ppm. ^{11}B{^{1}H} NMR (160.4 MHz, C$_6$D$_6$): δ = 54 (br, fwmh ≈ 1480 Hz, Pt−*B*), 32 (br, fwmh ≈ 470 Hz, N$_2$*B*Cl) ppm. ^{15}N NMR (50.7 MHz, C$_6$D$_6$, 297 K): δ = −247.2 (s, 2N, *Nt*Bu) ppm. ^{31}P{^{1}H} NMR (202.5 MHz, C$_6$D$_6$): δ = −12.8 (m + satellites, $^1J_{P\text{-}Pt}$ = 3198 Hz, $^3J_{P\text{-}Pt}$ = 79 Hz, 2P, *P$_2$*PtCl), −27.6 (m + satellites, $^1J_{P\text{-}Pt}$ = 2632 Hz, $^3J_{P\text{-}Pt}$ = 79 Hz, 2P, *P$_2$*PtB) ppm. Elemental analysis (%) calculated for [(C$_{18}$H$_{46}$B$_2$Cl$_2$N$_2$P$_4$Pt$_2$)·(C$_5$H$_{12}$)] (M$_W$ = 969.3): C 28.50, H 6.03, N 2.89; found: C 28.38, H 5.45, N 2.76. *Note: **4^{tBu}** slowly decomposed in solution at rt,*

*the major decomposition product being complex **5-Cl**. The fate of the [BNtBu]$_2$ fragment could not be determined as the ^{11}B NMR spectrum of the final product mixture was silent, and a colorless by-product insoluble in all common organic solvents was formed.*

[{*μ*-BNBN(SiMe$_3$)$_2$}{(*μ*-dmpm)PtBr}$_2$], 6

In a vial one equivalent of **1** (30.0 mg, 35.3 µmol) was dissolved in benzene (0.7 mL) and a 0.2 M solution of two equivalents of $BBr_2BN(SiMe_3)_2$ in benzene (23.4 mg, 0.35 mL, 70.5 µmol) was added. The mixture was then shaken, causing the immediate precipitation of a yellow solid. After filtration the solid was recrystallized from CH_2Cl_2/pentane and **6** (15.3 mg, 16.5 µmol, 46%) was isolated as a yellow solid. ^{1}H NMR (500.1 MHz, CD_2Cl_2): δ = 1.85–2.13 (m, 4H, P_2CH_2), 1.78 (br s + satellites, $^3J_{H\text{-}Pt}$ = 26 Hz, 12H, PCH_3), 1.63 (br s + satellites, $^3J_{H\text{-}Pt}$ = 42 Hz, 12H, PCH_3), 0.31 (s + satellites, $^2J_{H\text{-}Si}$ = 7 Hz, $^5J_{H\text{-}Pt}$ = 118 Hz, 18H, $Si(CH_3)_3$) ppm. ^{13}C{^{1}H} NMR (125.8 MHz, CD_2Cl_2): δ = 30.4 (t + satellites, $^1J_{13C\text{-}31P}$ = 30 Hz, P_2CH_2), 16.0 (dt + satellites, $^1J_{13C\text{-}31P}$ = 21 Hz, $^3J_{13C\text{-}31P}$ = 11 Hz, PCH_3), 15.6 (dt + satellites, $^1J_{13C\text{-}31P}$ = 19 Hz, $^3J_{13C\text{-}31P}$ = 9 Hz, PCH_3), 2.42 (s, $Si(CH_3)_3$) ppm. ^{11}B{^{1}H} NMR (160.5 MHz, CD_2Cl_2): δ = 56.7 (br, fwmh ≈ 1510 Hz), 26.3 (br, fwmh ≈ 690 Hz) ppm. ^{15}N NMR (50.7 MHz, C_6D_6, 297 K): *δ* = –337.8 (s, B*N*(SiMe$_3$)$_2$) ppm. *Note: the resonance for the BNB nitrogen could not be detected, presumably due to very strong quadrupolar coupling.* ^{31}P{^{1}H} NMR (202.5 MHz, CD_2Cl_2): δ = −7.1 (s + satellites higher order, $^1J_{P\text{-}Pt}$ = 3568 Hz, $^3J_{P\text{-}Pt}$ = 272 Hz, *Q* = 49 Hz, $^1J_{Pt\text{-}Pt}$ = 1826 Hz) ppm. Elemental analysis (%) calculated for $[(C_{16}H_{46}B_2Br_2N_2P_4Pt_2Si_2)\cdot(C_5H_{12})_{0.2}]$ (M_W = 1132.6): C 19.77, H 4.72, N 2.71%; found: C 19.47, H 4.65, N 2.35%. *Note: **6** slowly decomposed in solution at rt, the major decomposition product being the dibromodiplatinum complex [μ-(dmpm)PtBr]$_2$ (**5-Br**, δ(^{31}P) = –23.4 ppm, $^1J_{P\text{–}Pt}$ = 2640 Hz).[1] The fate of the BNBN(SiMe$_3$)$_2$ fragment could not be determined as the ^{11}B NMR spectrum of the final product mixture was silent, and a colorless by-product insoluble in all common organic solvents was formed.*

NMR spectra of new compounds

Note: because the complexes decomposed when exposed to vacuum, isolated compounds had to be dried at ambient pressure, which is why all NMR spectra contain residual crystallization solvent (pentane and/or hexane).

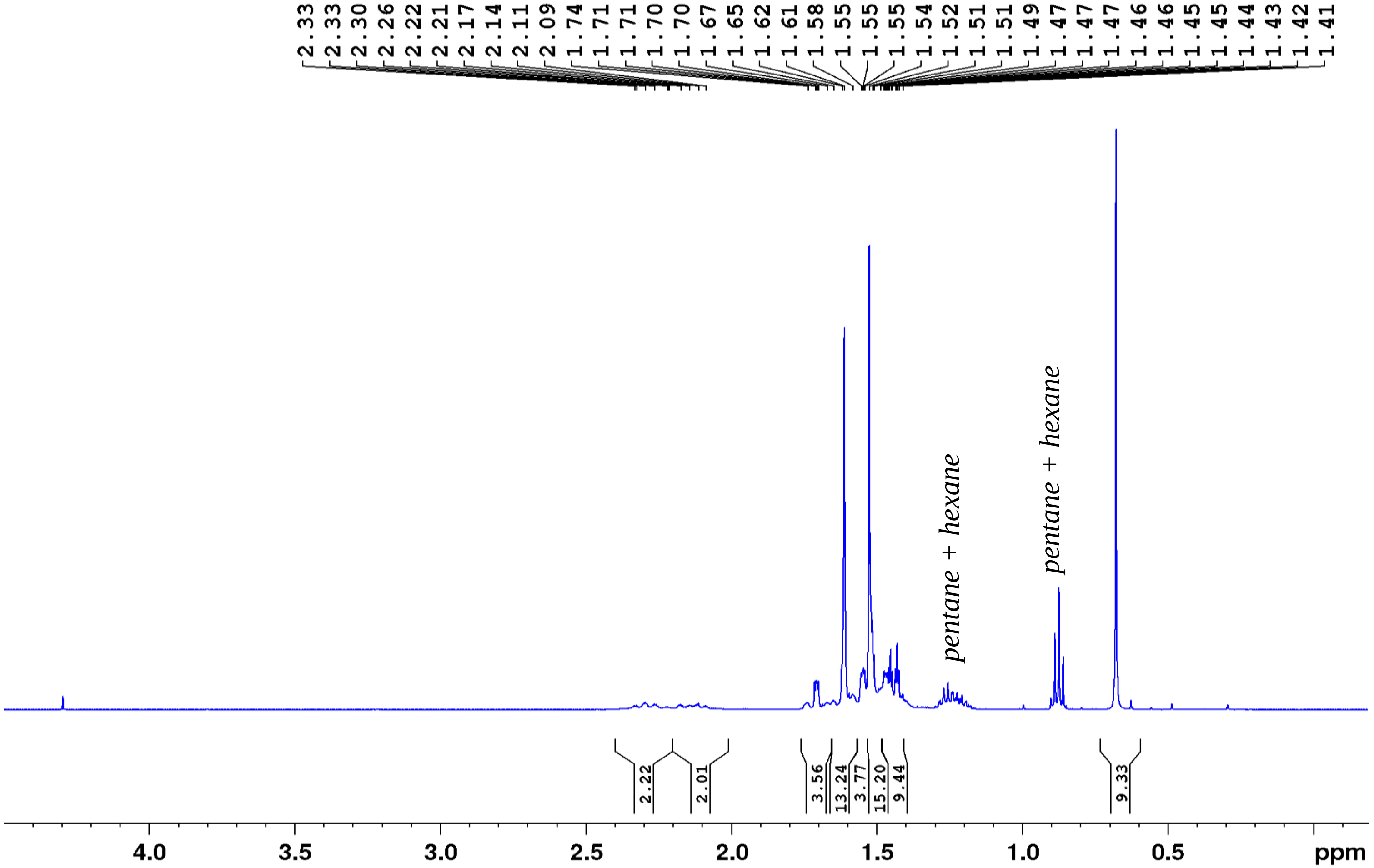

Figure S1. ^{1}H NMR spectrum **3*tBu*** in C_6D_6. Additional resonances at 0.87 (t) and 1.23 (m) ppm correspond to residual pentane and hexane.

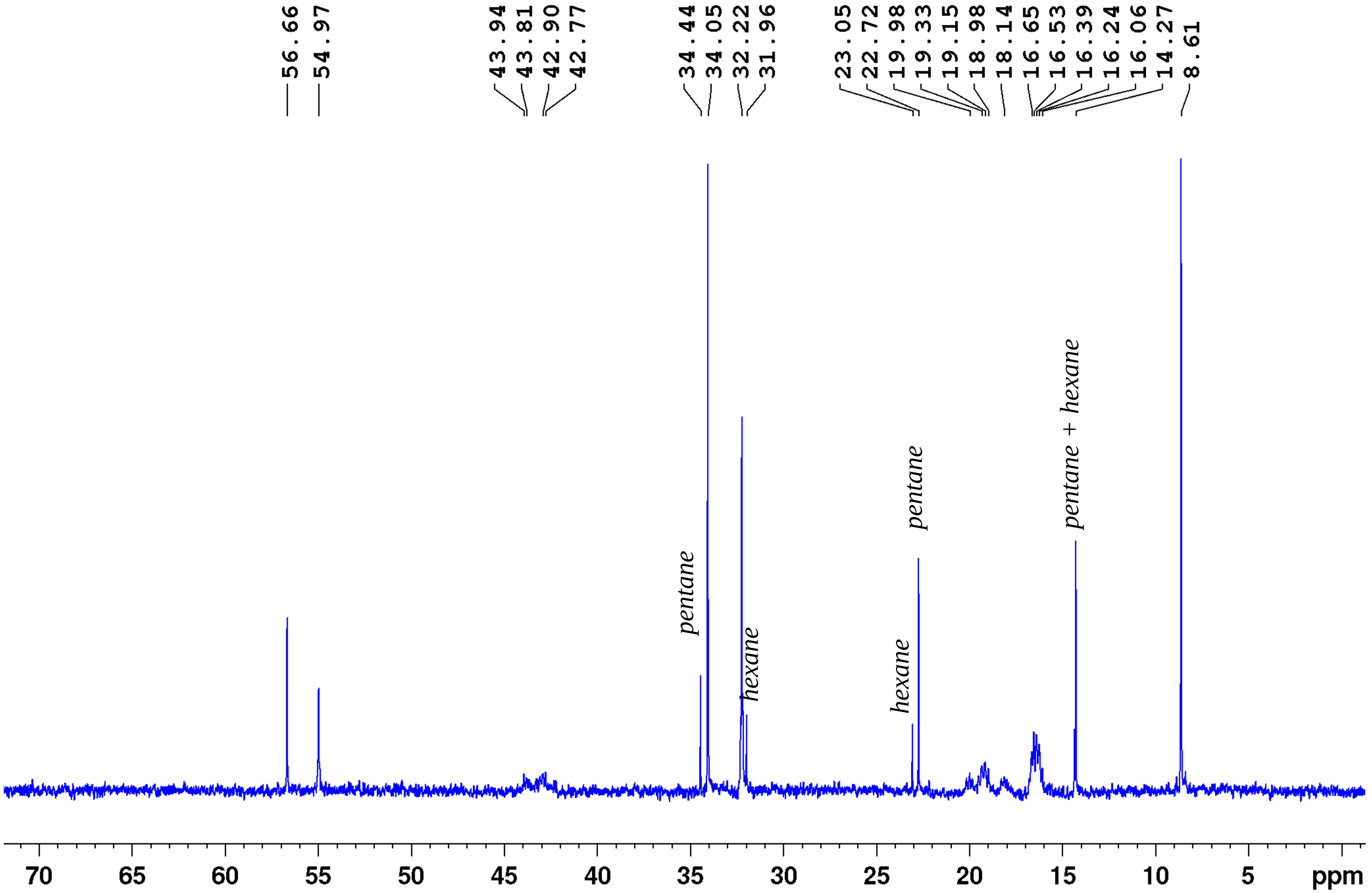

Figure S2. $^{13}C\{^{1}H\}$ NMR spectrum of $\mathbf{3^{\mathit{t}Bu}}$ in C_6D_6. Additional resonances at 14.3, 22.7 and 34.5 ppm correspond to residual pentane, those at 14.3, 23.0 and 32.0 to residual hexane.

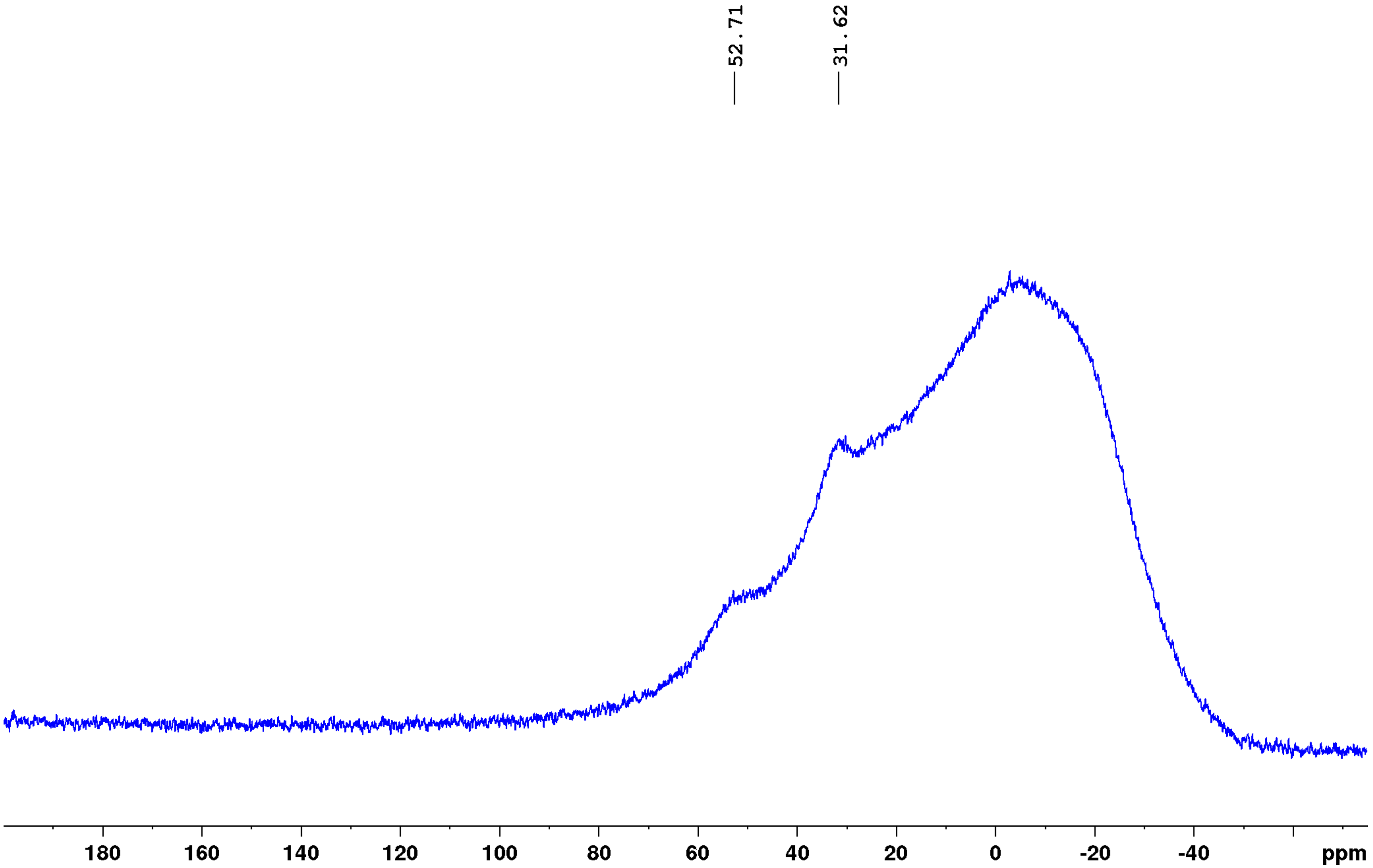

Figure S3. ^{11}B NMR spectrum of **3^{tBu}** in C_6D_6.

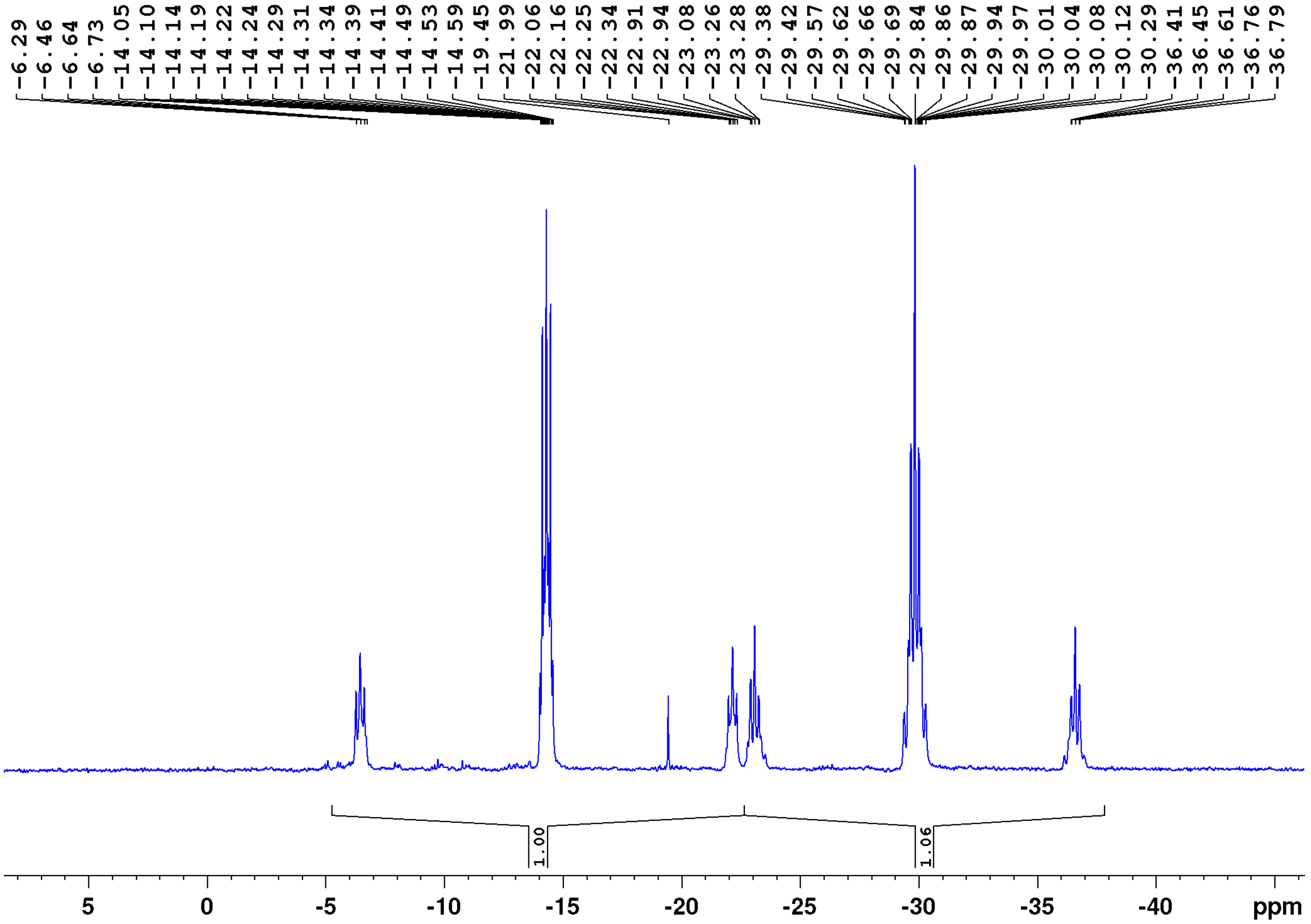

Figure S4. $^{31}P\{^{1}H\}$ NMR spectrum of $\mathbf{3}^{t\mathbf{Bu}}$ in C_6D_6. The additional resonance at −19.5 ppm corresponds to the decomposition product **5-Cl**.

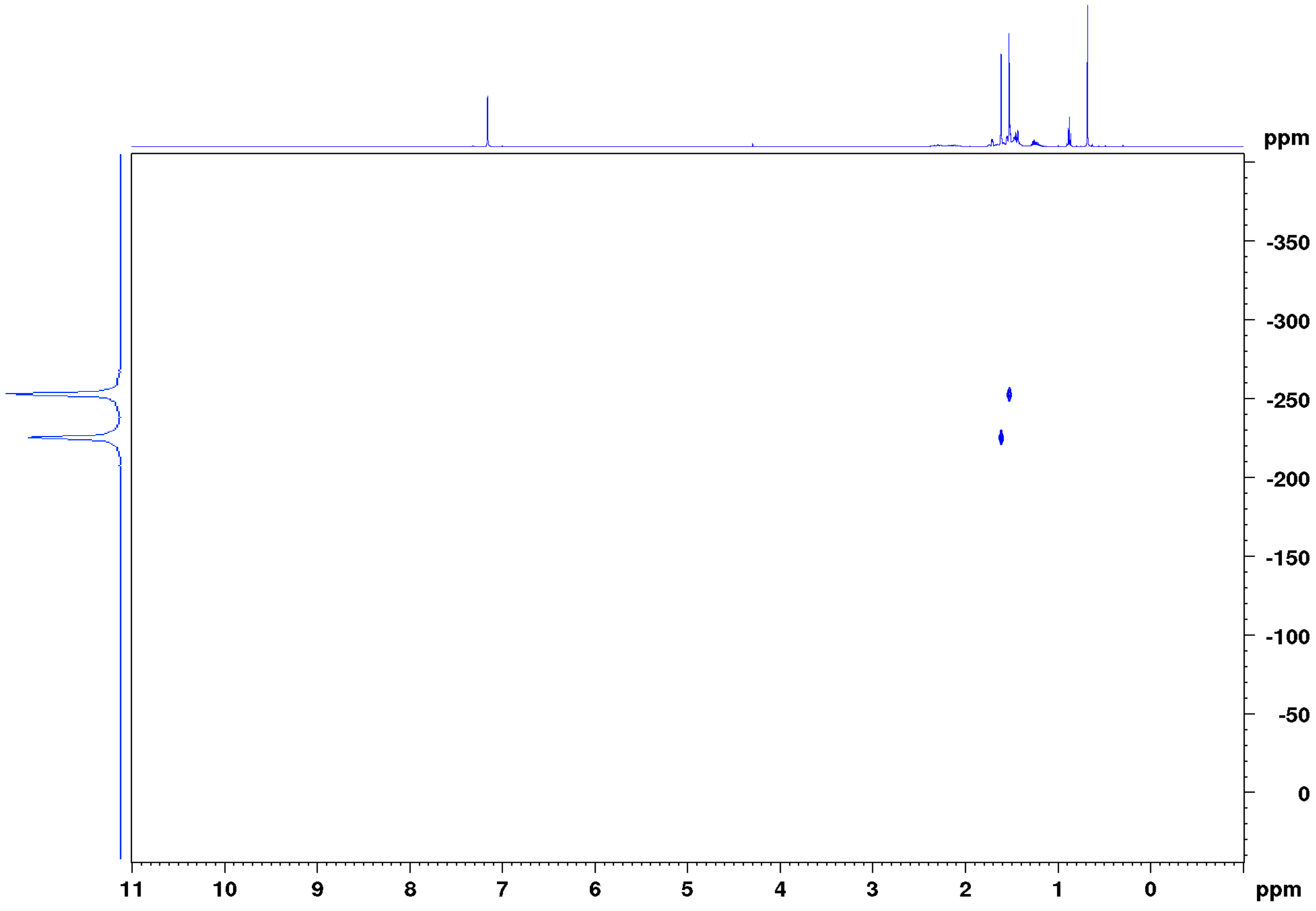

Figure S5. ^{15}N-^{1}H HMBC plot of **3^{tBu}** in C_6D_6.

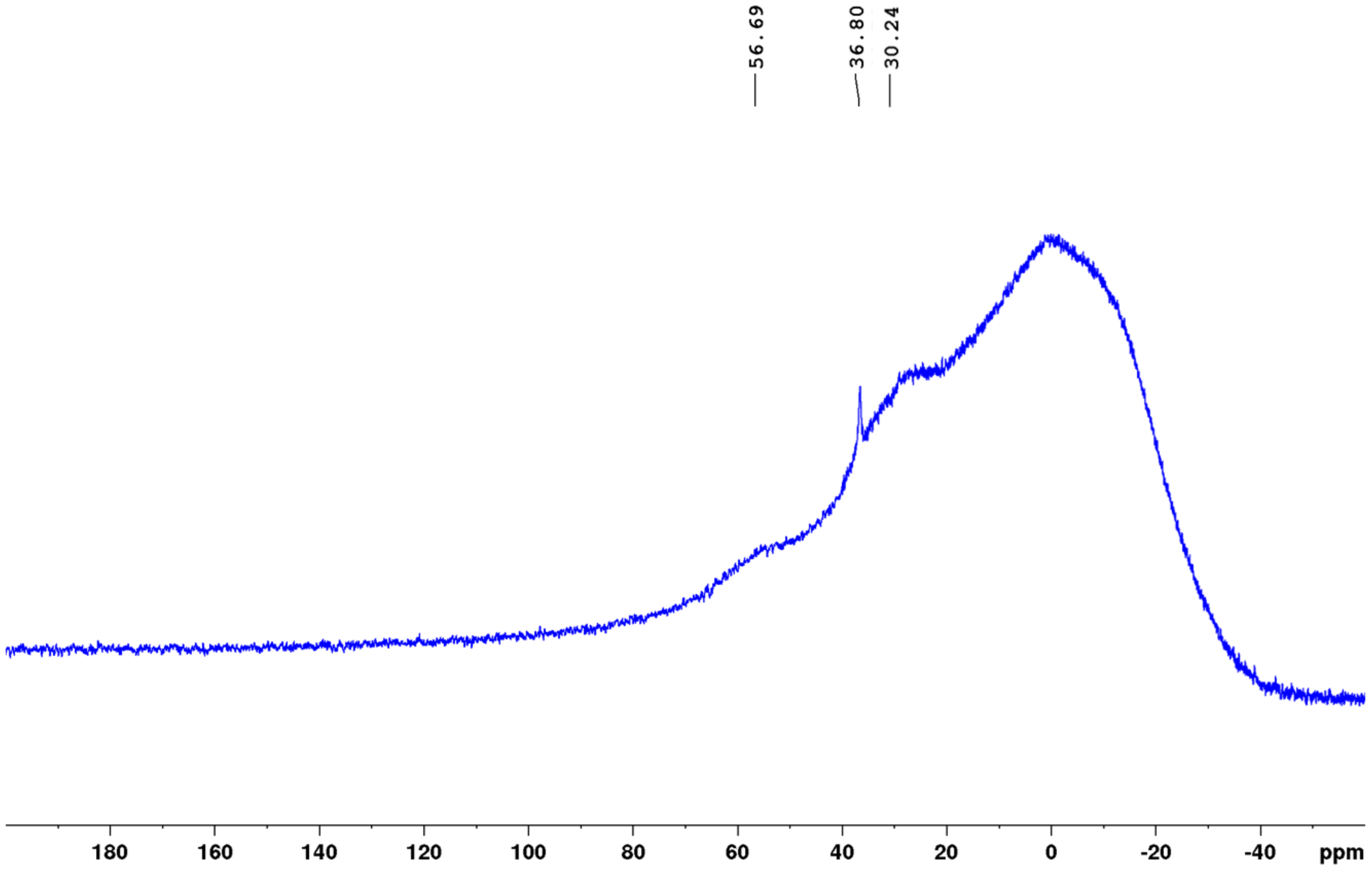

Figure S6. ^{11}B NMR spectrum of $\mathbf{3}^{\mathbf{SiMe3}}$ in CD_2Cl_2 generated *in situ*, prior to decomposition. The additional singlet at 36.8 ppm corresponds to excess $BCl_2N(SiMe_3)_2$.

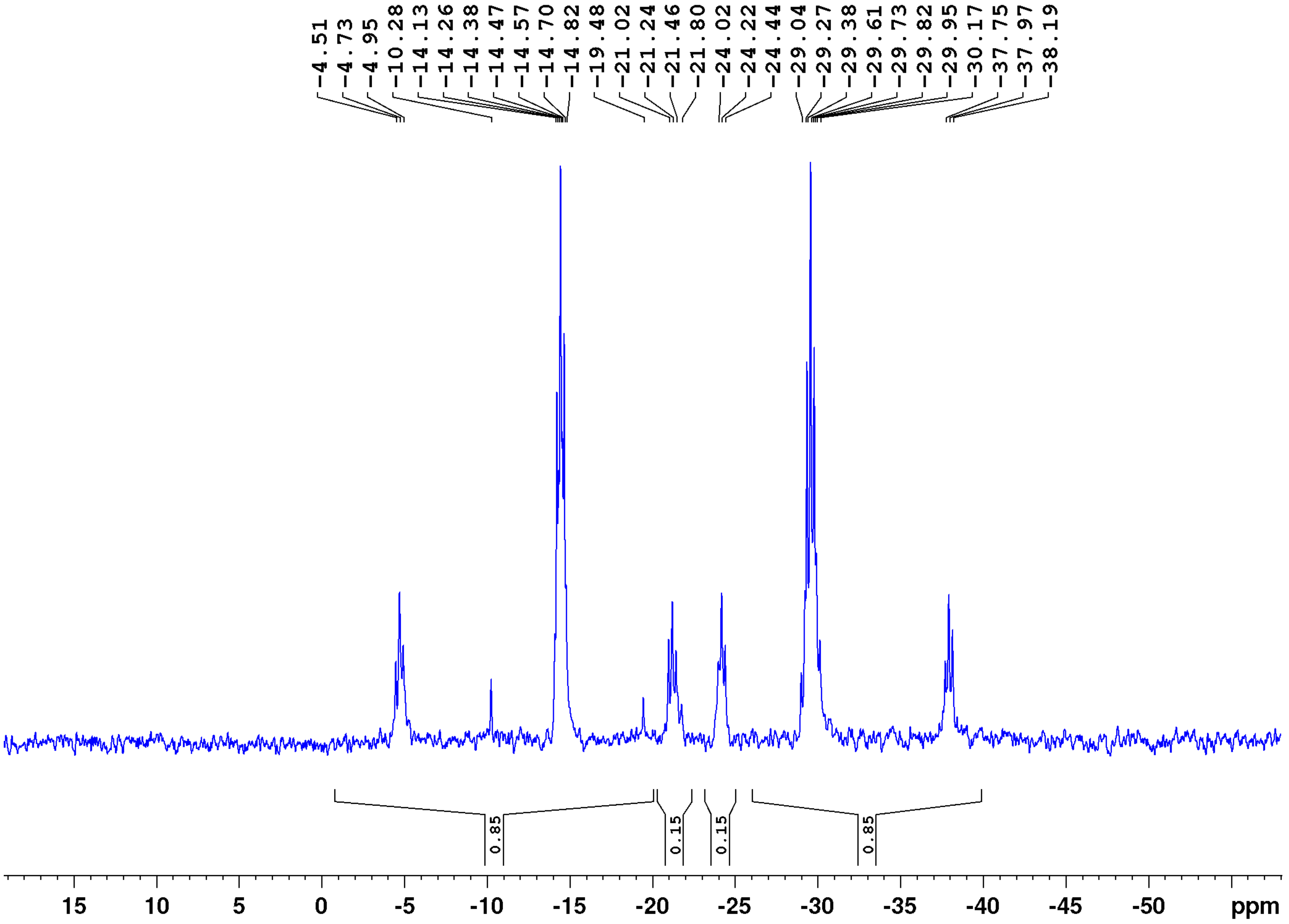

Figure S7. $^{31}P\{^{1}H\}$ NMR spectrum of $\mathbf{3^{SiMe3}}$ in CD_2Cl_2 generated *in situ*, prior to decomposition.

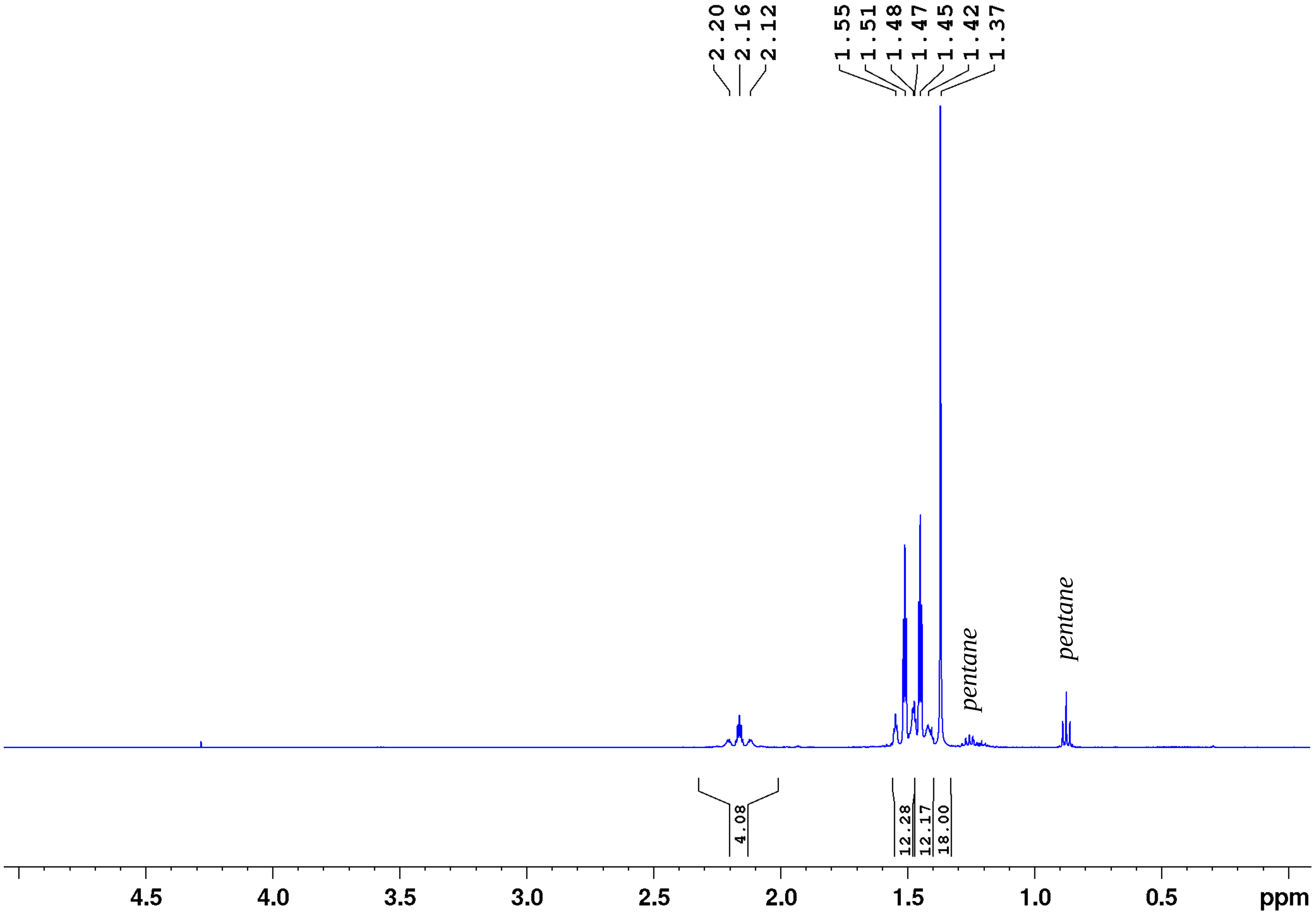

Figure S8. ^{1}H NMR spectrum of **4**$^{t\mathbf{Bu}}$ in C_6D_6. The additional resonances at 0.87 and 1.23 ppm correspond to residual pentane.

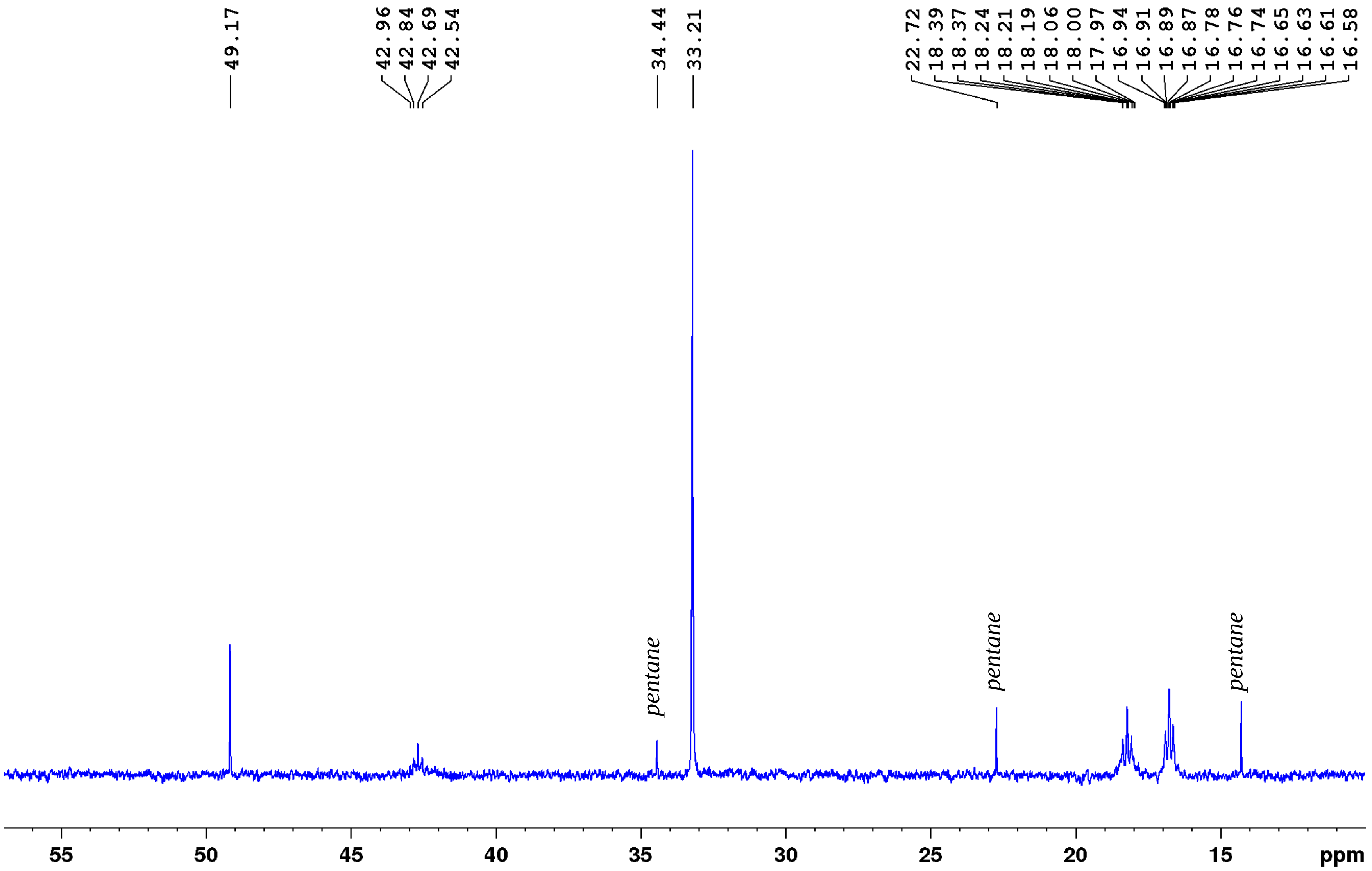

Figure S9. $^{13}C\{^{1}H\}$ NMR spectrum of $\mathbf{4}^{\mathbf{\mathit{t}Bu}}$ in C_6D_6. The additional resonances at 14.3, 22.7 and 34.5 ppm correspond to residual pentane.

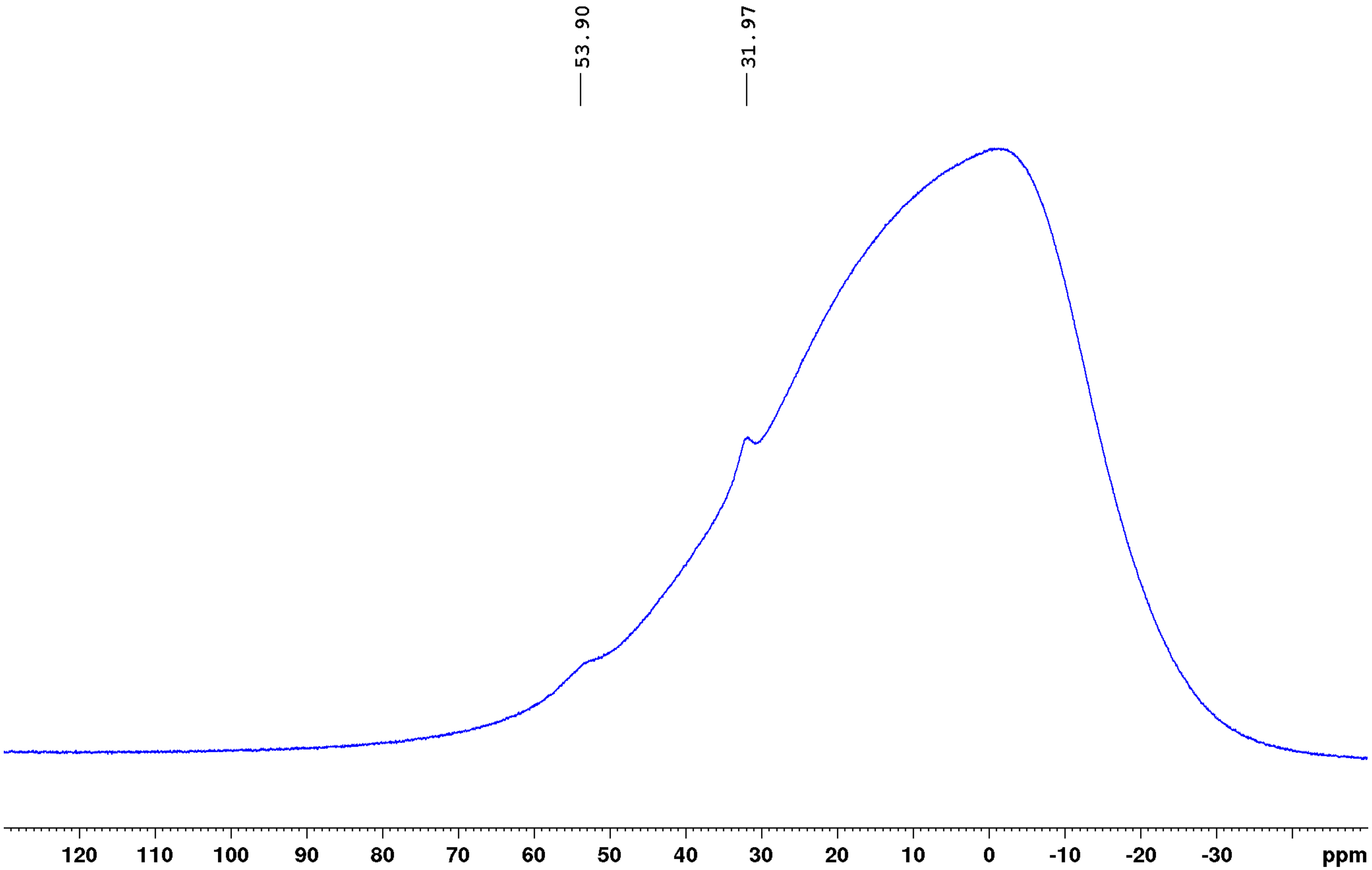

Figure S10. ^{11}B NMR spectrum of **4*tBu*** in C_6D_6.

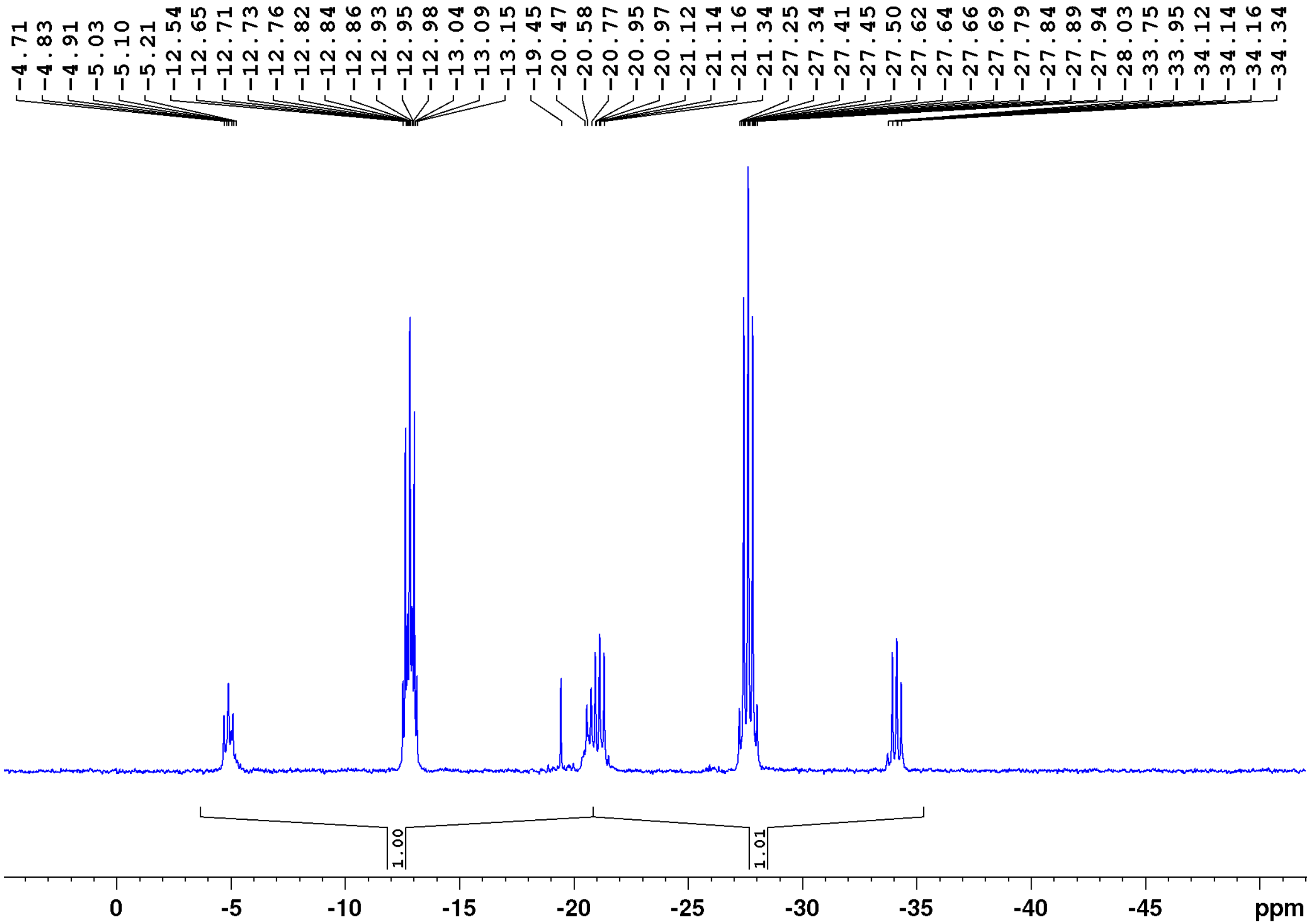

Figure S11. $^{31}P\{^{1}H\}$ NMR spectrum of $\mathbf{4}^{t\mathbf{Bu}}$ in C_6D_6. The additional resonance at −19.5 ppm corresponds to the decomposition product **5-Cl**.

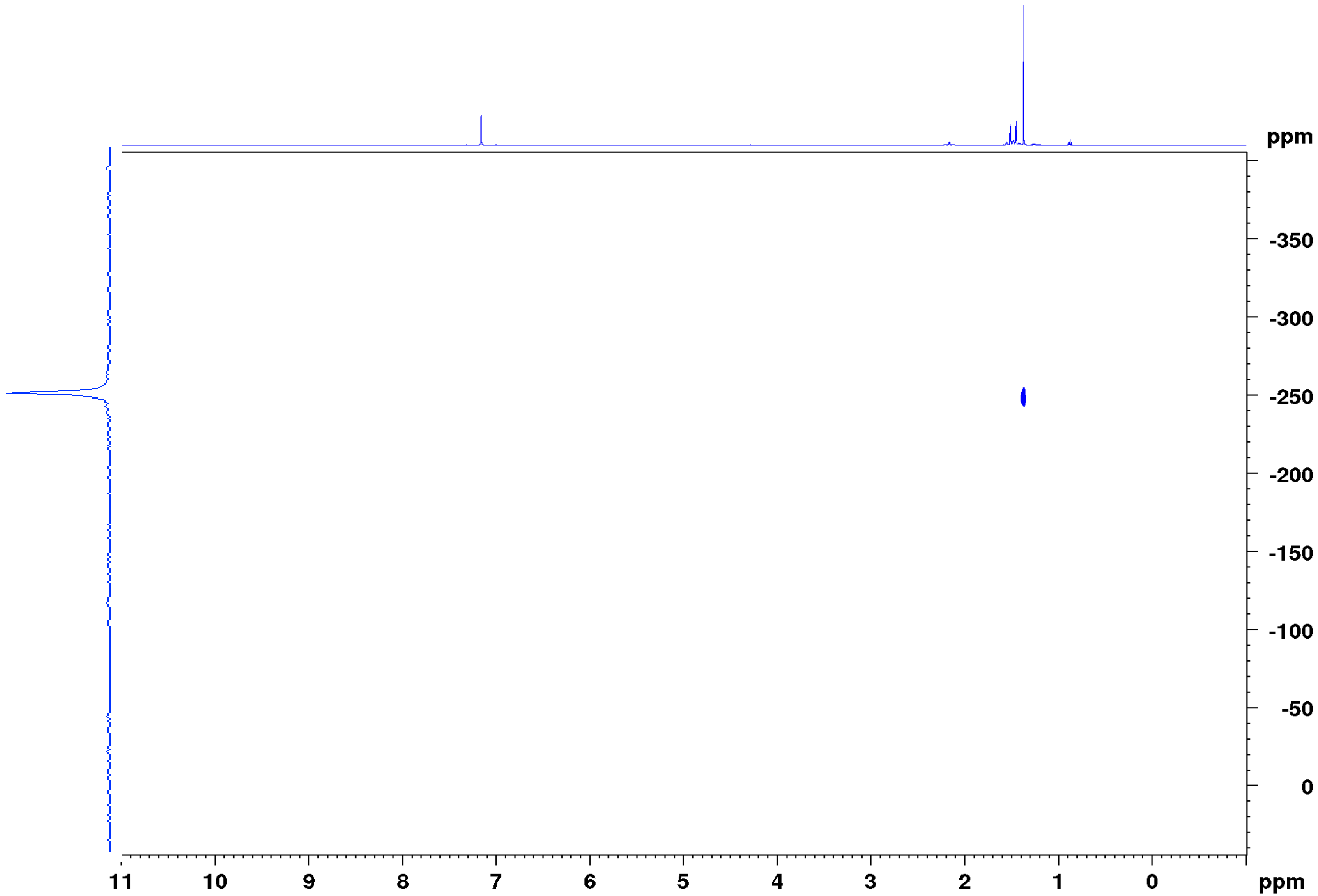

Figure S12. ^{15}N-^{1}H HMBC plot of **4**^{tBu} in C_6D_6.

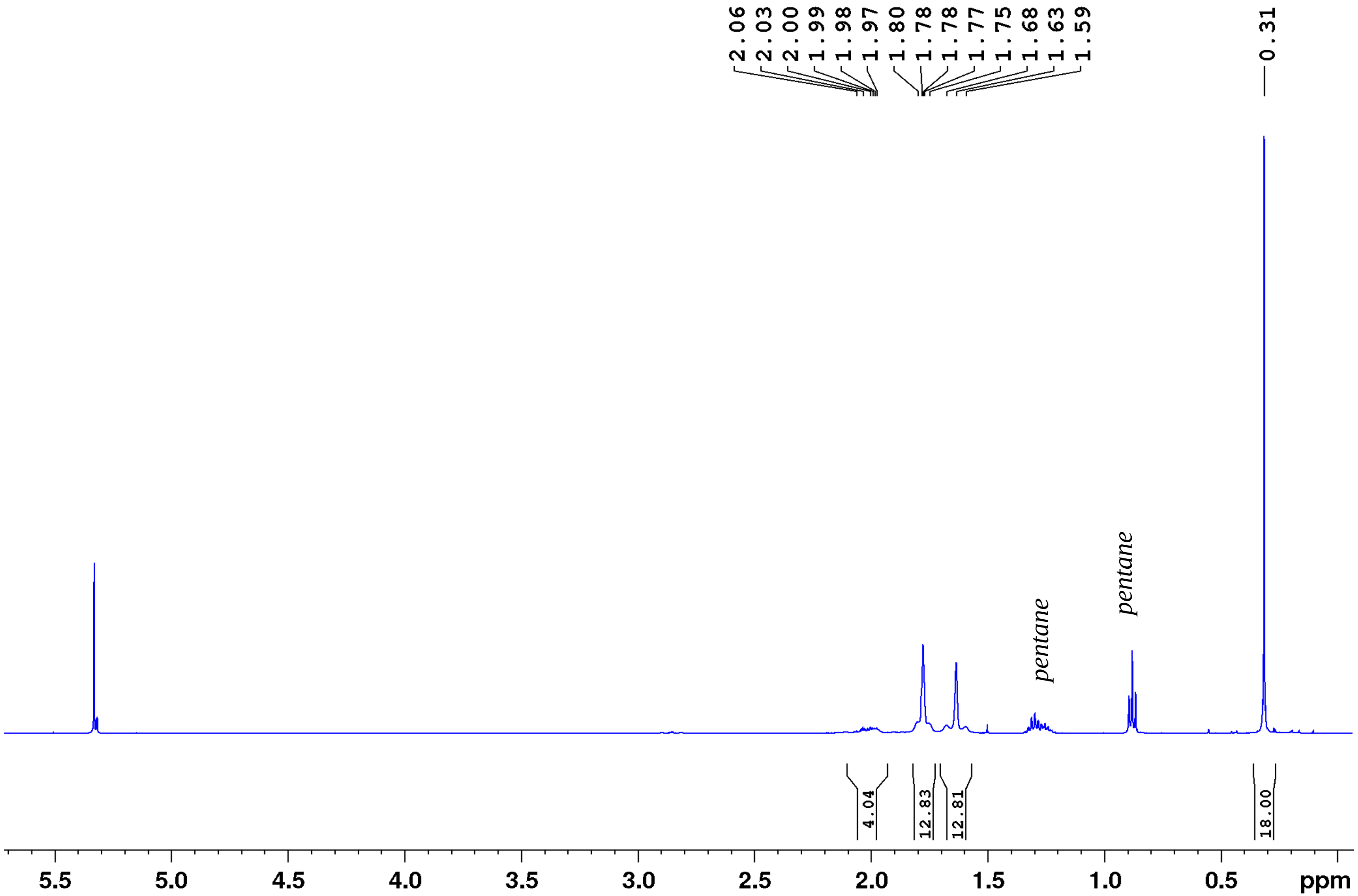

Figure S13. ^{1}H NMR spectrum of **6** in CD_2Cl_2. Additional resonances at 0.89 (t) and 1.30 (m) ppm correspond to residual pentane.

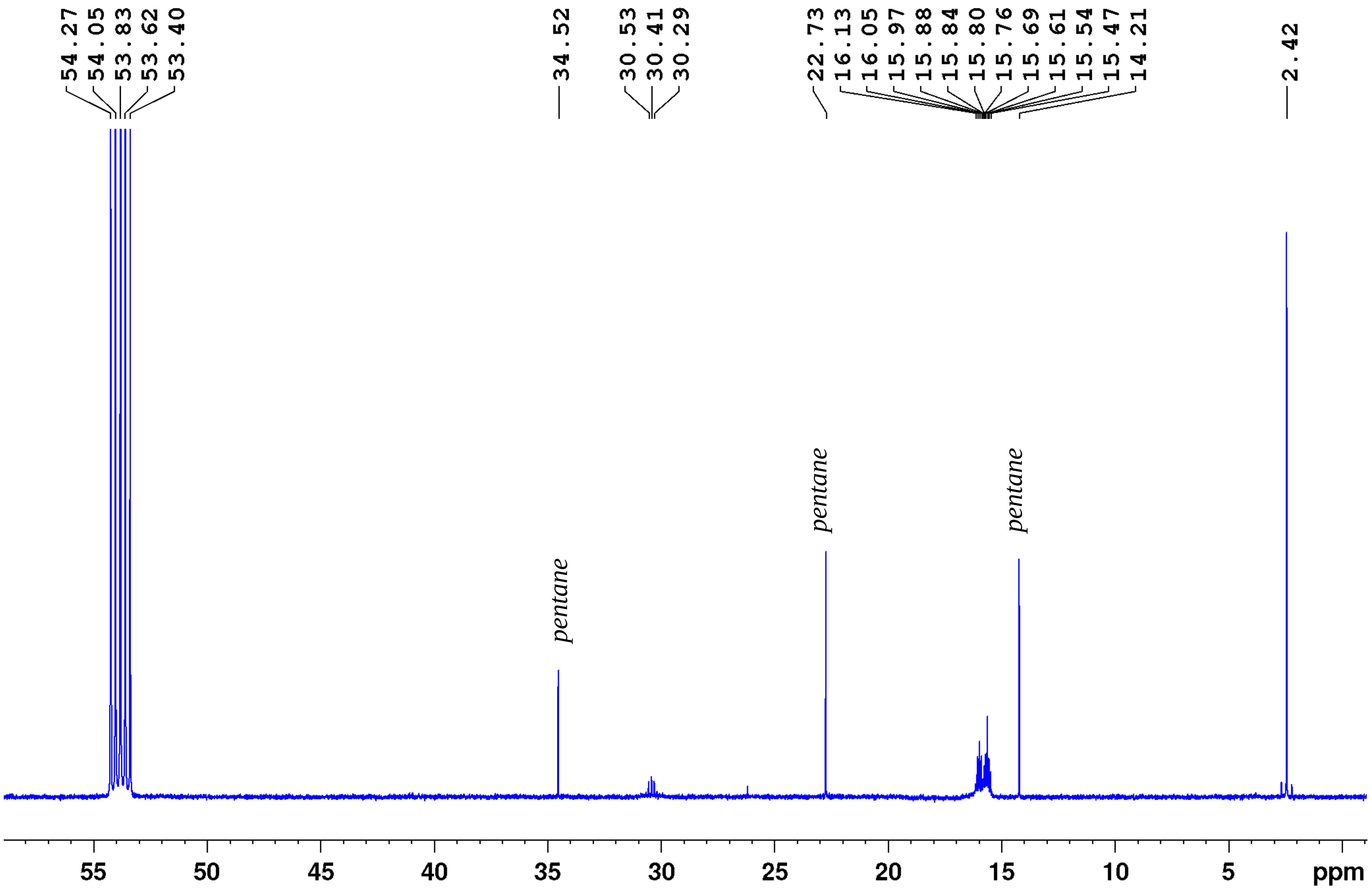

Figure S14. $^{13}C\{^{1}H\}$ NMR spectrum of **6** in CD_2Cl_2. Additional resonances at 14.24, 23.07 and 32.01 ppm correspond to residual pentane.

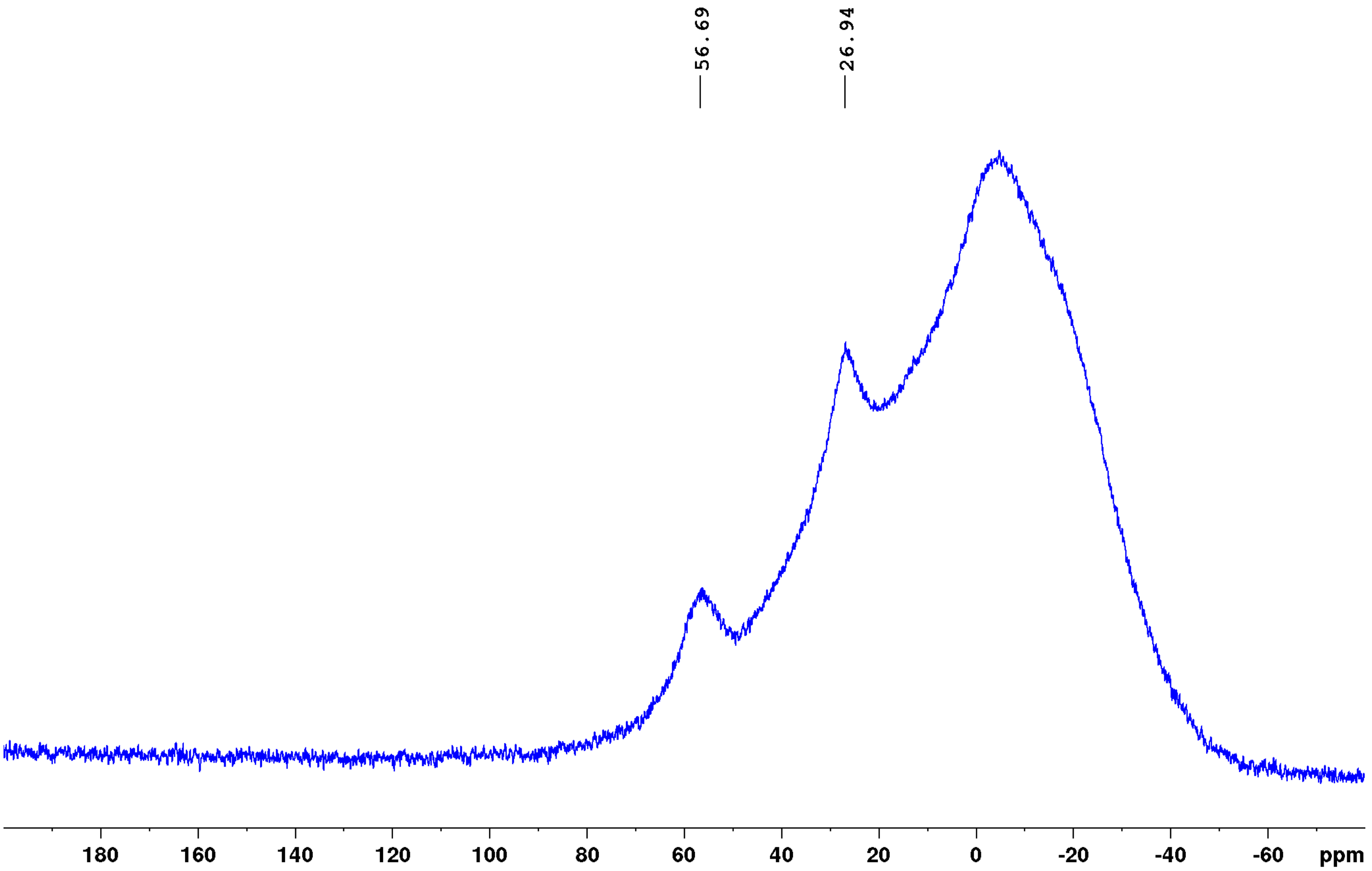

Figure S15. ^{11}B NMR spectrum of **6** in CD_2Cl_2.

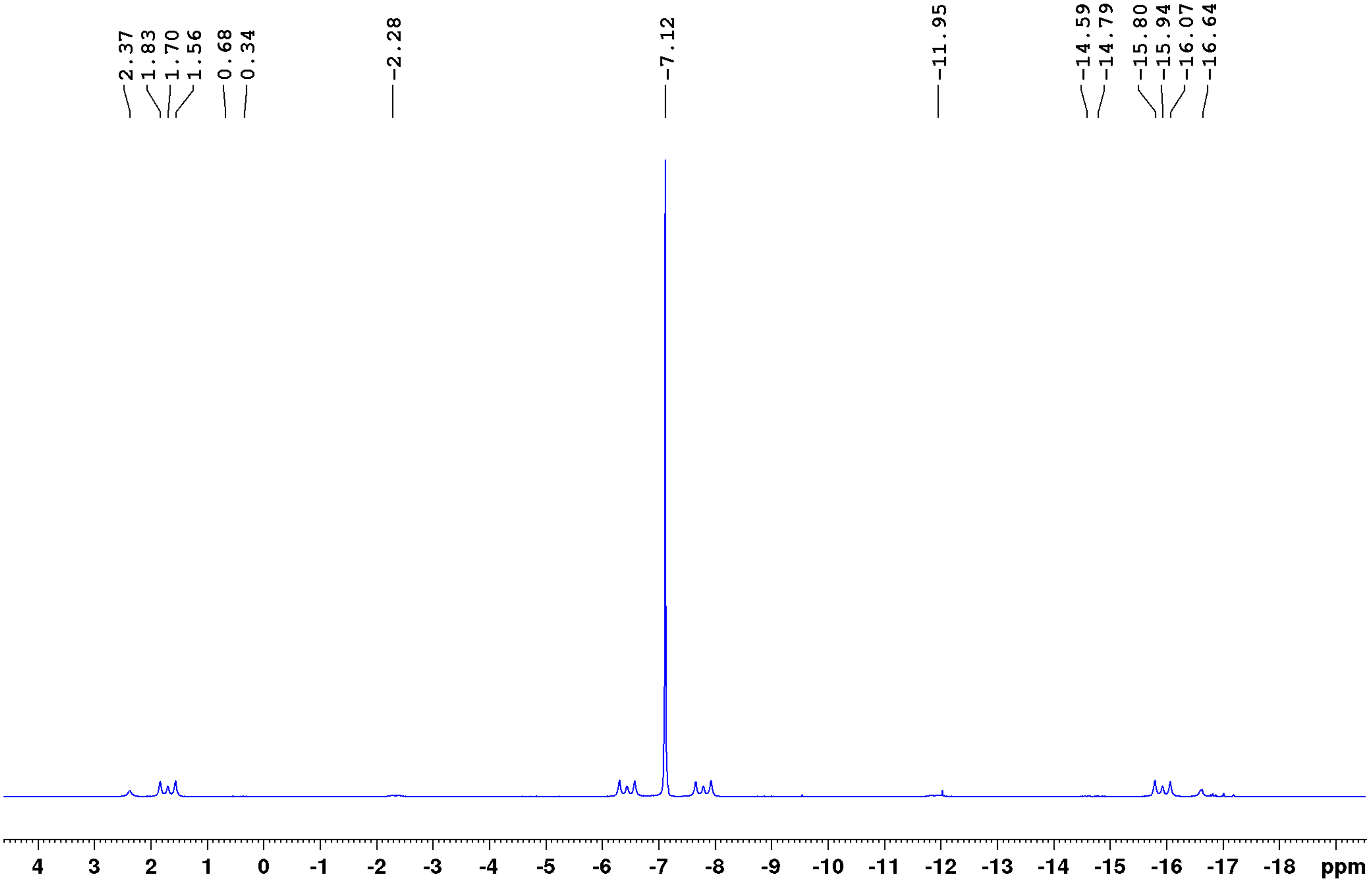

Figure S16. $^{31}P\{^1H\}$ NMR spectrum of **6** in CD_2Cl_2.

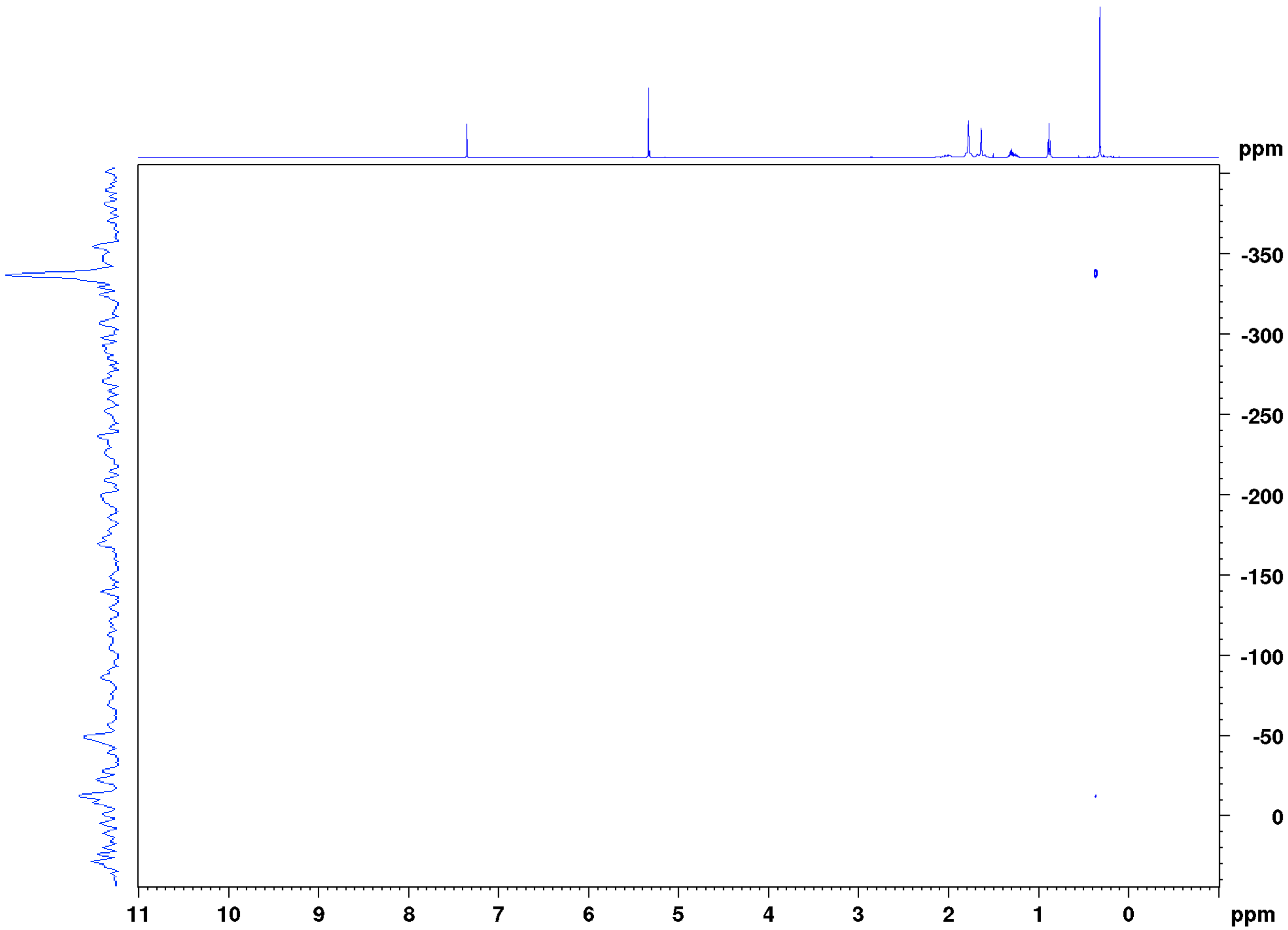

Figure S17. ^{15}N HMBC plot of **6** in C_6D_6.

Decomposition of 3^{SiMe3}

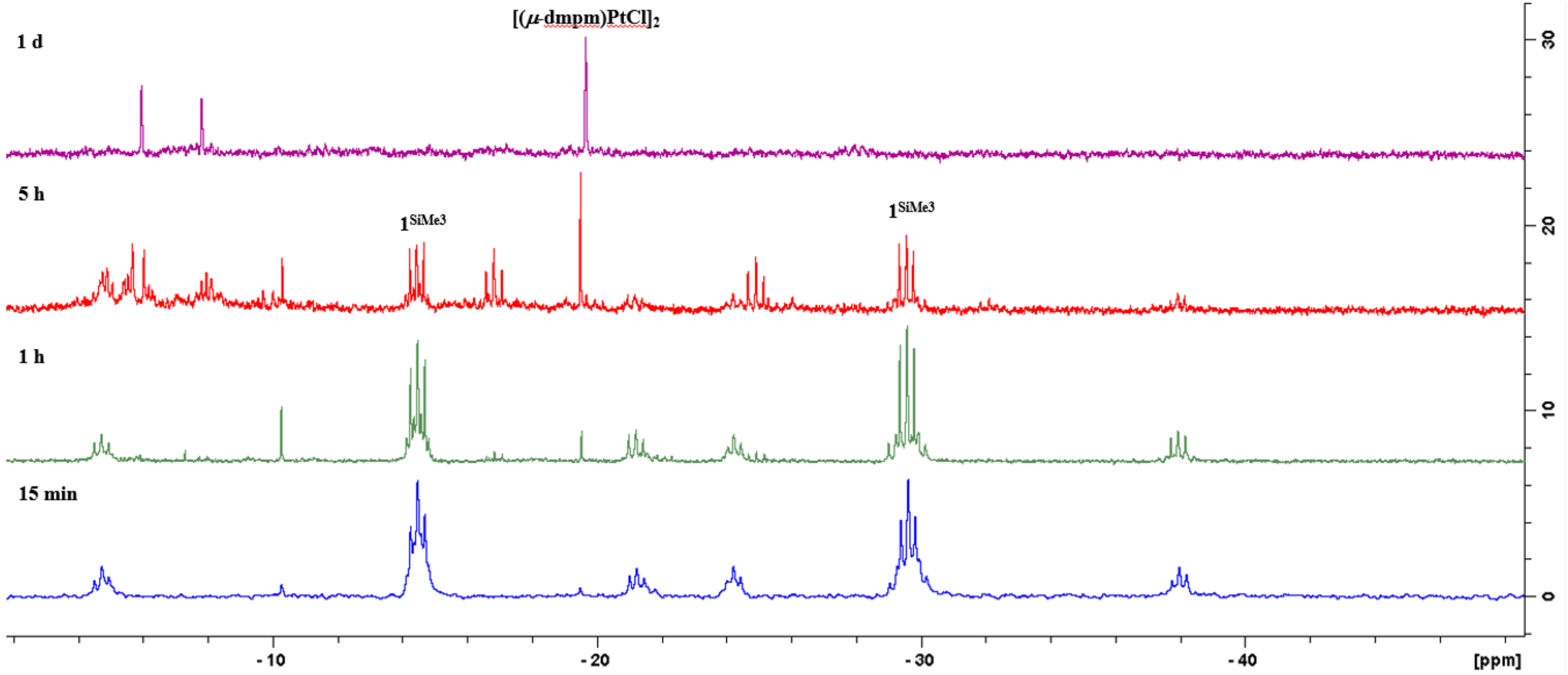

Figure S18. ^{31}P NMR-spectroscopic monitoring of a sample of $\mathbf{3}^{SiMe3}$ in CD_2Cl_2 at rt.

X-ray crystallographic data

All crystal data were collected on a BRUKER D8 QUEST diffractometer equipped with a CMOS area detector and multi-layer mirror monochromated $Mo_{K\alpha}$ radiation. Structures were solved using the intrinsic phasing method,[6] refined with the SHELXL program[7] and expanded using Fourier techniques. All non-hydrogen atoms were refined anisotropically. Hydrogen atoms were included in structure factor calculations. All hydrogen atoms were assigned to idealized geometric positions.

Crystallographic data have been deposited with the Cambridge Crystallographic Data Center as supplementary publication nos. CCDC-2081529 (**4**^{tBu}), 2081530 (**6**), 2081531 (**3**^{tBu}). These data can be obtained free of charge from The Cambridge Crystallographic Data Centre *via* www.ccdc.cam.ac.uk/data_request/cif.

Refinement details for 3^{tBu}: The asymmetric unit contains three benzene molecules, one of which was too heavily disordered to model accurately and was treated as a diffuse contribution to the overall scattering without specific atom positions by SQUEEZE/PLATON.[8] This removed 87 electrons from the unit cell void, corresponding to ca. two molecules of benzene (42 electrons each), i.e. one molecule per asymmetric unit. The second molecule of benzene was modelled as twofold disordered in a 42:58 ratio, with AFIX 66 and ADPs restrained with SIMU. The third molecule of benzene was not disordered. The asymmetric unit also contains two crystallographically distinct units of the complex, which present different degrees of disorder. First complex molecule: One of the dmpm ligands (RESI 8 and 81 PCP) was modelled as twofold disordered in a 56:44 ratio with ADPs restrained using SIMU 0.005 (no bond length restraints). The BClN(*t*Bu)($SiMe_3$) residue (RESI 4 and 41 BNBN2) was modelled as twofold flip-disordered in a 40:60 ratio with ADPs restrained using SIMU 0.005. Since no bond length restraints were applied, structural parameters of this molecular unit can be discussed. Second complex molecule: The BClN(*t*Bu)($SiMe_3$) residue (RESI 2, 21, 22 and 23 BNBN1) was modelled as fourfold disordered (twofold disordered by flipping the ligand 180° and each orientation twofold disordered by a slight rotation about the B1-N1 bond). The sum of the four corresponding FVAR was adjusted to 1.0 using SUMP, and refined to a 38:18:34:10 ratio.

ADPs within the disorder were restrained with SIMU 0.003 and 1,2- and 1,3-distances restrained to similarity with SAME 0.01. The N1_1 B2_2/21/22/23 distances were restrained to similarity with SADI 0.01. Since heavy bond length restraints were applied, structural parameters of this molecular unit cannot be discussed.

Crystal data for 3^{tBu}: $C_{21}H_{55}B_2Cl_3N_2P_4Pt_2Si \cdot C_6H_6$[+squeezed $(C_6H_6)_{0.5}$], M_r = 1083.89, colorless plate, 0.686×0.275×0.172 mm^3, triclinic space group $P\bar{1}$, a = 9.4607(16) Å, b = 20.222(6) Å, c = 24.964(5) Å, α = 108.858(13)°, β = 97.434(12)°, γ = 94.381(17)°, V = 4445.9(17) Å^3, Z = 4, ρ_{calcd} = 1.619 g·cm^{-3}, μ = 6.656 mm^{-1}, F(000) = 2112, T = 100(2) K, R_1 = 0.0402, wR^2 = 0.0690, 18914 independent reflections [$2\theta \leq 53.544°$] and 1240 parameters.

Refinement details for 4^{tBu}: The crystal data was solved as a pseudo-merohedral twin and the data refined with the keyword HKLF 5. The BASF parameter was refined to 42%. The asymmetric unit contains two half benzene molecules positioned around inversion centers. The first (RESI 5 BEN1) was not disordered but idealized with AFIX 66 and with ADPs restrained using ISOR 0.01 and SIMU 0.01. The second (RESI 6 and 16 BEN2) was modelled as twofold rotationally disordered, with parts refined to a 22:28 ratio, rings idealized with AFIX 66 and ADPs restrained with SIMU and ISOR 0.01. The $B(N(tBu))_2$ fragment was modelled as twofold disordered by a slight rotation about the Pt2-B1 bond. The two parts (RESI 4 and 14 B2N2) were refined to a 72:28 ratio. ADPs within the disordered parts were restrained with SIMU 0.005, except for Cl2 for which ADPs were restrained using SIMU 0.002. 1,2 and 1,3 distances within both parts were restrained to similarity with SAME_B2N2 0.01 B1 > C8. The B1-N and B2-N distances were each restrained to similarity with SADI 0.01. Since bond length restraints were applied, structural parameters of this fragment cannot be discussed.

Crystal data for 4^{tBu}: $C_{18}H_{46}B_2Cl_4N_2P_4Pt_2 \cdot C_6H_6$, M_r = 975.24, colorless plate, 0.122×0.165×0.299 mm^3, triclinic space group $P\bar{1}$, a = 11.5645(17) Å, b = 12.746(2) Å, c = 13.334(2) Å, α = 86.807(5)°, β = 74.749(5)°, γ = 74.253(5)°, V = 1824.7(7) Å^3, Z = 2, ρ_{calcd} = 1.775 g·cm^{-3}, μ = 7.996 mm^{-1}, F(000) = 940, T = 100(2) K, R_1 = 0.0708, wR^2 = 0.1161, 7163 independent reflections [$2\theta \leq 52.040°$] and 622 parameters.

Refinement details for 6: The asymmetric unit contains one *o*-difluorobenzene molecule, which was modelled as twofold disordered, the parts being refined to a 38:62 ratio. The benzene rings in both parts were idealized with AFIX 66 and all ADPs restrained with SIMU 0.01.

Crystal data for 6: $C_{16}H_{46}B_2Br_2F_2N_2P_4Pt_2Si_2\cdot C_6H_4F_2$, M_r = 1132.32, yellow block, 0.246×0.215×0.194 mm^3, monoclinic space group $P2_1/n$, a = 13.7751(4) Å, b = 11.6664(4) Å, c = 23.8278(8) Å, β = 96.2650(10)°, V = 3806.4(2) Å^3, Z = 4, ρ_{calcd} = 1.976 g·cm^{-3}, μ = 9.699 mm^{-1}, $F(000)$ = 2152, T = 100(2) K, R_1 = 0.0395, wR^2 = 0.0711, 8104 independent reflections [$2\theta \leq 53.562°$] and 406 parameters.

Computational details

Geometry optimizations and Hessian calculations were performed for **6** using the M06,[9] M062X,[9] PBE0,[10] ωB97XD,[11] B3LYP,[12] and B3PW91[12]c,[13] functionals in association with the cc-pVDZ[14],aug-cc-pVDZ-PP{Pt}[15] basis set. Dispersion corrections were incorporated using the zero-damped D3 model[16] for M06 and M062X and the Becke-Johnson-damped variant D3(BJ)[17] for PBE0, B3LYP, and B3PW91. Additional computations of the butatriene C_4H_4 molecule and the parent system H_2BNBNH_2 were done at the M06-D3/cc-pVDZ,aug-cc-pVDZ-PP{Pt} level of theory. The truncated model systems of **6**, namely **mod-1**, **mod-2**, **mod-3**, and **mod-4** (see Figure S19), were optimized at the ωB97XD/cc-pVDZ,aug-cc-pVDZ-PP{Pt} level. All optimized structures were characterized as minimum energy geometries as only positive eigenvalues were obtained in the vibrational frequency calculations. Mayer bond orders (MBOs)[18] were obtained for both systems at the M06-D3/cc-pVDZ,aug-cc-pVDZ-PP{Pt} level of theory using the Multiwfn program. Intrinsic bond orbitals (IBOs)[19] were computed with IBOView. All DFT calculations were done in Gaussian 16, Revision B.01.[20]

Table S1. Comparison of the optimized B1-N1-B2 and N1-B2-N2 angles of **6** using distinct DFT functionals. All optimizations were carried out with the cc-pVDZ,aug-cc-pVDZ-PP{Pt} basis set.

DFT functional	B1-N1-B2	N1-B2-N2
M06-D3	161.3	176.2
M062X-D3	160.1	178.1
PBE0-D3(BJ)	157.9	175.5
ωB97XD	156.3	175.8
B3LYP-D3(BJ)	155.2	174.8
B3PW91-D3(BJ)	152.5	175.2

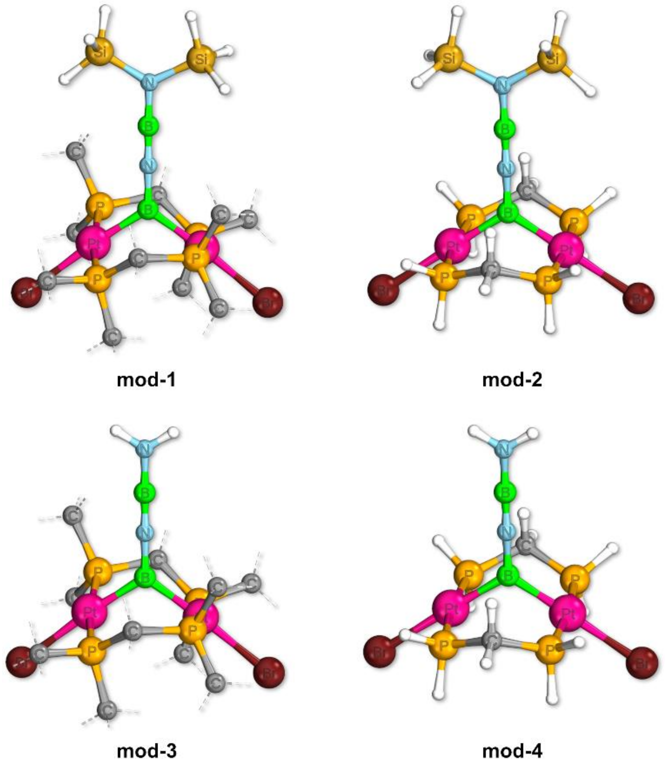

Figure S19. Optimized structures of the truncated models of **6** investigated herein at the ωB97XD/cc-pVDZ,aug-cc-pVDZ-PP{Pt} level of theory.

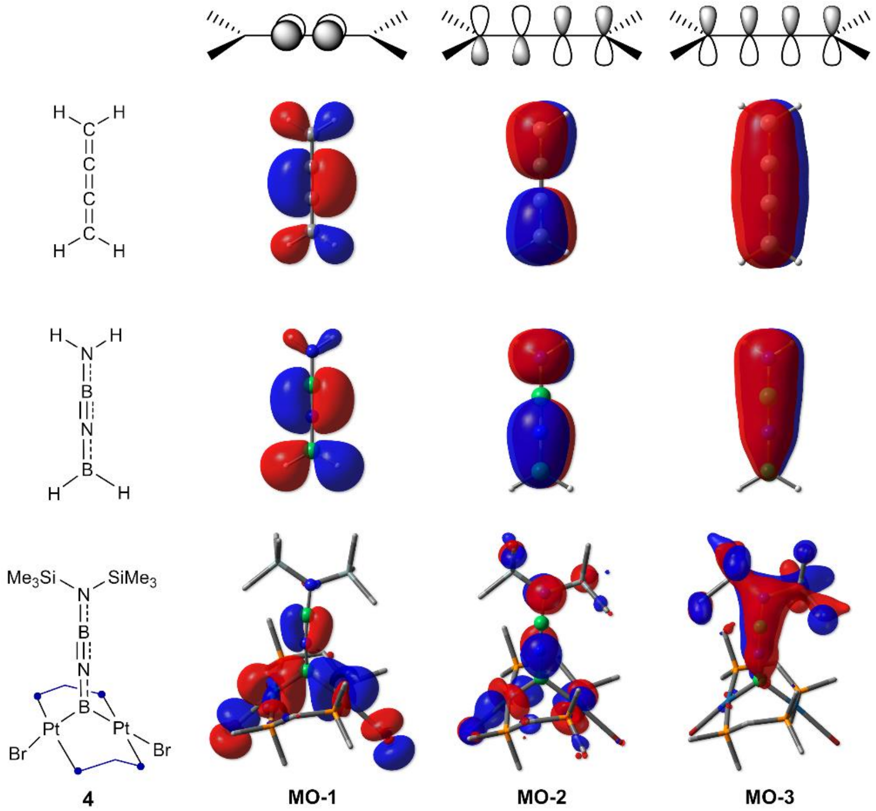

Figure S20. Comparison of selected molecular orbitals of **6** and those of the parent systems C_4H_4 and H_2BNBNH_2 highlighting the cumulenic character of their C_4 and BNBN motifs.

Cartesian coordinates

**6** – M06-D3/cc-pVDZ,aug-cc-pVDZ-PP{Pt}			
Br	2.759425000	3.548832000	0.259008000
Pt	0.940373000	1.650840000	-0.110319000
C	-0.368768000	0.305997000	2.832170000
H	-1.377155000	-0.066988000	2.577677000
H	-0.333195000	0.476669000	3.923932000
P	-0.132932000	1.912850000	1.922897000
Br	2.674665000	-3.484096000	0.682467000
Pt	0.916098000	-1.586745000	0.076268000
C	1.187407000	-0.251760000	-3.130725000
H	1.740150000	-0.408161000	-4.075263000
H	0.170016000	0.101023000	-3.378163000
P	1.010640000	-1.862890000	-2.216555000
P	1.973816000	1.102754000	-2.129926000
P	0.808532000	-1.043389000	2.342133000
B	-0.274011000	0.025380000	-0.357036000
N	-1.617488000	-0.003569000	-0.739635000
Si	-5.255316000	1.325312000	-0.916435000
C	-6.544605000	0.878593000	-2.202294000
H	-6.069635000	0.428387000	-3.089109000
H	-7.080905000	1.785267000	-2.528861000
H	-7.292427000	0.168732000	-1.815289000
B	-2.891384000	-0.030746000	-0.677747000
N	-4.259930000	-0.121684000	-0.544992000
Si	-4.755462000	-1.581114000	0.389638000
C	-6.078063000	1.919880000	0.663956000
H	-6.737540000	1.151523000	1.101373000
H	-6.697730000	2.809153000	0.460979000
H	-5.326688000	2.198617000	1.421749000
C	-3.618576000	-2.977664000	-0.123551000
H	-3.684162000	-3.166184000	-1.208056000
H	-3.888393000	-3.908999000	0.401175000

H	-2.565455000	-2.744022000	0.118247000
C	-6.551956000	-1.969567000	0.022842000
H	-7.226390000	-1.145410000	0.308694000
H	-6.858084000	-2.859698000	0.597002000
H	-6.703736000	-2.190186000	-1.046278000
C	-4.495097000	-1.191579000	2.208715000
H	-3.437498000	-0.915822000	2.369686000
H	-4.712889000	-2.063947000	2.846773000
H	-5.121665000	-0.351930000	2.550955000
C	-4.086470000	2.625763000	-1.590825000
H	-3.294384000	2.888107000	-0.869676000
H	-4.643798000	3.545273000	-1.834535000
H	-3.590772000	2.276594000	-2.511912000
C	0.669065000	2.986167000	3.171423000
H	0.067785000	3.033674000	4.093788000
H	0.793465000	3.989593000	2.736457000
H	1.677417000	2.604202000	3.390249000
C	-1.855715000	2.550480000	1.881934000
H	-2.449121000	1.898557000	1.219903000
H	-1.851362000	3.568908000	1.463344000
H	-2.301688000	2.563864000	2.890417000
C	2.424611000	-0.451736000	2.965423000
H	2.367439000	-0.173834000	4.031251000
H	2.751716000	0.410006000	2.359192000
H	3.150806000	-1.267034000	2.820978000
C	0.343692000	-2.384955000	3.499180000
H	1.019342000	-3.234036000	3.310796000
H	-0.687663000	-2.705458000	3.281782000
H	0.422164000	-2.058927000	4.549149000
C	2.378104000	-2.879287000	-2.887451000
H	2.322693000	-3.877646000	-2.427089000
H	3.343074000	-2.440958000	-2.592639000
H	2.310005000	-2.952096000	-3.985058000
C	-0.476573000	-2.600322000	-2.995614000

H	-0.393974000	-2.612963000	-4.094542000
H	-1.355642000	-2.014819000	-2.684262000
H	-0.588930000	-3.629194000	-2.619624000
C	3.685909000	0.511923000	-1.860949000
H	3.673474000	-0.339109000	-1.159242000
H	4.243853000	1.335155000	-1.387551000
H	4.165293000	0.222744000	-2.811323000
C	2.166093000	2.441976000	-3.363448000
H	2.760372000	2.107934000	-4.229483000
H	2.666669000	3.283198000	-2.858564000
H	1.171916000	2.776542000	-3.696240000

6 – M062X-D3/cc-pVDZ,aug-cc-pVDZ-PP{Pt}

Br	0.948567000	4.222521000	0.140315000
Pt	0.246354000	1.625798000	-0.002118000
C	0.018718000	-0.168539000	2.959232000
H	-0.783696000	-0.893528000	2.745826000
H	0.090504000	-0.034407000	4.050243000
P	-0.461583000	1.434013000	2.149423000
Br	4.020007000	-2.494956000	0.323435000
Pt	1.589686000	-1.380990000	0.026957000
C	0.803138000	-0.050960000	-3.071939000
H	1.198301000	0.023127000	-4.097475000
H	-0.290777000	-0.175349000	-3.123041000
P	1.482837000	-1.565989000	-2.234216000
P	1.090704000	1.528089000	-2.139156000
P	1.574406000	-0.920378000	2.281054000
B	-0.112307000	-0.347345000	-0.152612000
N	-1.406797000	-0.859523000	-0.391166000
Si	-4.919648000	0.489368000	-0.781656000
C	-6.093449000	0.191912000	-2.217908000
H	-5.558391000	-0.269748000	-3.061407000
H	-6.503949000	1.154781000	-2.560520000
H	-6.937066000	-0.456834000	-1.944024000

B	-2.676188000	-0.966760000	-0.397910000
N	-4.058515000	-1.044979000	-0.381874000
Si	-4.780304000	-2.510913000	0.366857000
C	-5.878808000	1.059384000	0.732488000
H	-6.627625000	0.313944000	1.042263000
H	-6.413234000	1.995588000	0.508288000
H	-5.205438000	1.245935000	1.582562000
C	-3.724761000	-3.971816000	-0.149871000
H	-3.751493000	-4.103918000	-1.242095000
H	-4.087544000	-4.898572000	0.319811000
H	-2.676085000	-3.821504000	0.151583000
C	-6.547585000	-2.706833000	-0.234771000
H	-7.182885000	-1.854949000	0.050944000
H	-6.981596000	-3.611864000	0.217925000
H	-6.584628000	-2.820995000	-1.328298000
C	-4.734345000	-2.292581000	2.233691000
H	-3.692853000	-2.176692000	2.573361000
H	-5.163854000	-3.166214000	2.747668000
H	-5.298018000	-1.399543000	2.543470000
C	-3.604235000	1.729759000	-1.284499000
H	-2.807336000	1.847290000	-0.531924000
H	-4.061849000	2.714861000	-1.464087000
H	-3.118968000	1.405260000	-2.219237000
C	0.132161000	2.710940000	3.319589000
H	-0.274679000	2.542472000	4.327047000
H	-0.173303000	3.690491000	2.926274000
H	1.230031000	2.698167000	3.340234000
C	-2.281747000	1.451698000	2.397884000
H	-2.725454000	0.656596000	1.780165000
H	-2.667098000	2.424825000	2.060720000
H	-2.541707000	1.293457000	3.454844000
C	2.879833000	0.294354000	2.695935000
H	2.872388000	0.545250000	3.767057000
H	2.730428000	1.197702000	2.084624000

H	3.839244000	-0.162559000	2.412861000
C	1.881931000	-2.320402000	3.419548000
H	2.811042000	-2.803564000	3.084621000
H	1.058112000	-3.041924000	3.332745000
H	1.976003000	-1.976385000	4.459407000
C	3.064229000	-1.870646000	-3.104638000
H	3.506707000	-2.784196000	-2.683946000
H	3.758029000	-1.046239000	-2.892343000
H	2.903634000	-1.970460000	-4.187779000
C	0.377060000	-2.889011000	-2.855222000
H	0.303785000	-2.868045000	-3.952006000
H	-0.611634000	-2.742443000	-2.398525000
H	0.782067000	-3.855639000	-2.525117000
C	2.910900000	1.721672000	-2.117914000
H	3.343098000	0.942493000	-1.471140000
H	3.120155000	2.703596000	-1.668908000
H	3.335129000	1.663520000	-3.131256000
C	0.511918000	2.807291000	-3.313866000
H	1.054552000	2.748505000	-4.268146000
H	0.676470000	3.781321000	-2.831063000
H	-0.565922000	2.676954000	-3.482823000

6 – PBE0-D3(BJ)/cc-pVDZ,aug-cc-pVDZ-PP{Pt}

Br	3.691228000	2.760198000	0.244179000
Pt	1.450878000	1.485579000	-0.164318000
C	-0.424137000	0.733833000	2.635961000
H	-1.449857000	0.691100000	2.236481000
H	-0.479022000	0.930959000	3.718920000
P	0.434212000	2.131738000	1.768161000
Br	1.394550000	-3.898210000	0.879701000
Pt	0.431004000	-1.591755000	0.108127000
C	1.228622000	-0.504681000	-3.073693000
H	1.722434000	-0.833104000	-4.002744000
H	0.357039000	0.114797000	-3.340641000

P	0.589649000	-1.969233000	-2.132701000
P	2.344150000	0.590497000	-2.081511000
P	0.322370000	-0.929326000	2.304516000
B	-0.170390000	0.291785000	-0.400724000
N	-1.489635000	0.612129000	-0.753433000
Si	-5.148480000	2.010789000	-0.919305000
C	-6.243308000	1.584351000	-2.385111000
H	-5.630519000	1.267128000	-3.243305000
H	-6.830899000	2.464813000	-2.691412000
H	-6.947058000	0.773100000	-2.147350000
B	-2.762302000	0.639753000	-0.669647000
N	-4.133949000	0.583769000	-0.511481000
Si	-4.674117000	-0.916402000	0.349442000
C	-6.191382000	2.474827000	0.575610000
H	-6.880360000	1.667997000	0.869061000
H	-6.799779000	3.364373000	0.346136000
H	-5.551916000	2.713429000	1.439754000
C	-3.475427000	-2.288400000	-0.086400000
H	-3.556151000	-2.547633000	-1.153438000
H	-3.709584000	-3.191881000	0.499557000
H	-2.423089000	-2.014482000	0.116131000
C	-6.419557000	-1.347342000	-0.198386000
H	-7.146524000	-0.556881000	0.042344000
H	-6.742385000	-2.264785000	0.319653000
H	-6.458134000	-1.539250000	-1.281611000
C	-4.634555000	-0.571503000	2.199162000
H	-3.618118000	-0.284794000	2.512811000
H	-4.922362000	-1.470519000	2.767826000
H	-5.319873000	0.243410000	2.477375000
C	-3.985341000	3.415378000	-1.363949000
H	-3.376507000	3.724229000	-0.500360000
H	-4.567147000	4.289045000	-1.698845000
H	-3.298587000	3.130800000	-2.176441000
C	1.494146000	2.857851000	3.068328000

H	0.898763000	3.157049000	3.944004000
H	2.016372000	3.722165000	2.634623000
H	2.261600000	2.128355000	3.359038000
C	-0.911587000	3.356614000	1.570339000
H	-1.610488000	2.962498000	0.819444000
H	-0.474820000	4.290574000	1.188749000
H	-1.432506000	3.548079000	2.520640000
C	1.991522000	-0.832670000	3.040430000
H	1.956756000	-0.507641000	4.091361000
H	2.599068000	-0.139442000	2.439236000
H	2.432701000	-1.837031000	2.961188000
C	-0.614258000	-2.021906000	3.433054000
H	-0.208718000	-3.036179000	3.302156000
H	-1.672258000	-2.027852000	3.134210000
H	-0.521773000	-1.700706000	4.481428000
C	1.678484000	-3.338312000	-2.663856000
H	1.331531000	-4.255227000	-2.166810000
H	2.700675000	-3.146289000	-2.312206000
H	1.665958000	-3.454741000	-3.757953000
C	-0.989707000	-2.323174000	-2.988389000
H	-0.848430000	-2.414010000	-4.075829000
H	-1.690064000	-1.507593000	-2.760783000
H	-1.396018000	-3.261928000	-2.585721000
C	3.788181000	-0.460224000	-1.695273000
H	3.474221000	-1.240552000	-0.985753000
H	4.525867000	0.184166000	-1.195218000
H	4.219532000	-0.910384000	-2.602291000
C	2.965232000	1.779521000	-3.322978000
H	3.482897000	1.270330000	-4.149714000
H	3.653483000	2.457457000	-2.796804000
H	2.122619000	2.368692000	-3.711482000

6 – ωB97XD/cc-pVDZ,aug-cc-pVDZ-PP{Pt}

Br	3.772914000	2.800429000	0.291424000

Pt	1.542386000	1.457273000	-0.109974000
C	-0.452734000	0.671802000	2.656825000
H	-1.461985000	0.709846000	2.216617000
H	-0.542047000	0.847976000	3.740734000
P	0.522164000	2.042665000	1.865162000
Br	1.006351000	-4.119558000	0.813851000
Pt	0.350629000	-1.663935000	0.099559000
C	1.257265000	-0.483200000	-3.070567000
H	1.739080000	-0.798624000	-4.009882000
H	0.391974000	0.151898000	-3.320584000
P	0.594719000	-1.964863000	-2.166753000
P	2.397317000	0.591871000	-2.079088000
P	0.177258000	-1.040071000	2.320974000
B	-0.093793000	0.277980000	-0.382436000
N	-1.386190000	0.668171000	-0.787858000
Si	-4.950534000	2.271488000	-0.922345000
C	-6.102600000	1.969238000	-2.375091000
H	-5.533316000	1.634632000	-3.256477000
H	-6.629127000	2.899838000	-2.642306000
H	-6.860843000	1.205769000	-2.146307000
B	-2.654556000	0.755786000	-0.715160000
N	-4.029146000	0.769753000	-0.576764000
Si	-4.665555000	-0.720620000	0.225348000
C	-5.929916000	2.752558000	0.610065000
H	-6.661849000	1.979743000	0.892518000
H	-6.485974000	3.685981000	0.426617000
H	-5.258178000	2.918802000	1.467044000
C	-3.566237000	-2.157916000	-0.259728000
H	-3.622574000	-2.344025000	-1.343692000
H	-3.892837000	-3.073833000	0.258720000
H	-2.506017000	-1.983394000	-0.001425000
C	-6.436769000	-1.012224000	-0.331169000
H	-7.106754000	-0.181287000	-0.061281000
H	-6.822947000	-1.922141000	0.155948000

H	-6.490405000	-1.163056000	-1.420519000
C	-4.604191000	-0.448710000	2.086875000
H	-3.574767000	-0.221508000	2.407320000
H	-4.932874000	-1.352923000	2.624474000
H	-5.248864000	0.387592000	2.397897000
C	-3.711157000	3.614644000	-1.352153000
H	-3.057145000	3.854390000	-0.499726000
H	-4.246821000	4.534757000	-1.636258000
H	-3.069000000	3.320315000	-2.197114000
C	1.603137000	2.635191000	3.217724000
H	1.013878000	2.929309000	4.098938000
H	2.186569000	3.484399000	2.835104000
H	2.315436000	1.842995000	3.484557000
C	-0.742078000	3.358894000	1.702493000
H	-1.458126000	3.036018000	0.933587000
H	-0.244787000	4.276222000	1.356748000
H	-1.260392000	3.548715000	2.654233000
C	1.806431000	-1.091635000	3.152223000
H	1.739751000	-0.770387000	4.202576000
H	2.505076000	-0.448337000	2.596770000
H	2.170007000	-2.127547000	3.090573000
C	-0.907151000	-2.072481000	3.374077000
H	-0.571803000	-3.113746000	3.260354000
H	-1.940631000	-1.999775000	3.006222000
H	-0.862359000	-1.765816000	4.429582000
C	1.693284000	-3.327595000	-2.702079000
H	1.324814000	-4.255657000	-2.242582000
H	2.706074000	-3.150500000	-2.316457000
H	1.714614000	-3.415052000	-3.798520000
C	-0.970839000	-2.292825000	-3.060539000
H	-0.810657000	-2.370914000	-4.146109000
H	-1.667328000	-1.472483000	-2.836554000
H	-1.394310000	-3.231840000	-2.676242000
C	3.849574000	-0.472540000	-1.751567000

H	3.557925000	-1.257314000	-1.037818000
H	4.613902000	0.157770000	-1.274611000
H	4.243442000	-0.920426000	-2.676404000
C	2.989341000	1.800468000	-3.318601000
H	3.474840000	1.302254000	-4.170768000
H	3.699783000	2.466480000	-2.807656000
H	2.138442000	2.401561000	-3.668891000

6 – B3LYP-D3(BJ)/cc-pVDZ,aug-cc-pVDZ-PP{Pt}

Br	3.811946000	2.687237000	0.286422000
Pt	1.504045000	1.463308000	-0.195230000
C	-0.501783000	0.852027000	2.577746000
H	-1.514510000	0.837849000	2.146927000
H	-0.583826000	1.080103000	3.652632000
P	0.443291000	2.202409000	1.702994000
Br	1.188936000	-3.949086000	1.010880000
Pt	0.355113000	-1.578286000	0.122718000
C	1.357191000	-0.622150000	-3.068777000
H	1.887327000	-1.001588000	-3.957268000
H	0.525411000	0.017012000	-3.404552000
P	0.613071000	-2.038383000	-2.111641000
P	2.468369000	0.472341000	-2.052736000
P	0.194727000	-0.855614000	2.316392000
B	-0.154013000	0.319127000	-0.463824000
N	-1.449436000	0.669368000	-0.860684000
Si	-5.122368000	2.083843000	-0.952466000
C	-6.232155000	1.666897000	-2.415499000
H	-5.625983000	1.367124000	-3.285877000
H	-6.832843000	2.545798000	-2.704199000
H	-6.925146000	0.844623000	-2.181423000
B	-2.720567000	0.709206000	-0.760953000
N	-4.088651000	0.656450000	-0.573606000
Si	-4.633793000	-0.850296000	0.287775000
C	-6.154372000	2.517522000	0.564739000

H	-6.836699000	1.701956000	0.851257000
H	-6.769631000	3.409121000	0.358240000
H	-5.507184000	2.742897000	1.427723000
C	-3.445814000	-2.233322000	-0.158589000
H	-3.506665000	-2.464795000	-1.233963000
H	-3.705611000	-3.147757000	0.400417000
H	-2.393129000	-1.983456000	0.072011000
C	-6.389998000	-1.268292000	-0.251160000
H	-7.110572000	-0.473822000	-0.002537000
H	-6.716109000	-2.187115000	0.264296000
H	-6.437569000	-1.453935000	-1.335903000
C	-4.579310000	-0.502812000	2.141228000
H	-3.564623000	-0.195354000	2.442079000
H	-4.842506000	-1.406476000	2.716225000
H	-5.276587000	0.300089000	2.426826000
C	-3.975581000	3.510720000	-1.388928000
H	-3.356612000	3.807529000	-0.527406000
H	-4.568745000	4.387065000	-1.698895000
H	-3.297469000	3.247481000	-2.216736000
C	1.493379000	2.917149000	3.029917000
H	0.880015000	3.254006000	3.880041000
H	2.062105000	3.753213000	2.599705000
H	2.220524000	2.162890000	3.358837000
C	-0.855734000	3.478113000	1.435843000
H	-1.543664000	3.097077000	0.668373000
H	-0.372193000	4.389528000	1.054634000
H	-1.402099000	3.705325000	2.364579000
C	1.849301000	-0.793485000	3.110137000
H	1.789726000	-0.429940000	4.147873000
H	2.500701000	-0.141412000	2.509544000
H	2.257216000	-1.814236000	3.081928000
C	-0.822206000	-1.885468000	3.449685000
H	-0.446635000	-2.916062000	3.367774000
H	-1.868657000	-1.866016000	3.112223000

H	-0.757515000	-1.530700000	4.490056000
C	1.681405000	-3.467883000	-2.547878000
H	1.276184000	-4.357629000	-2.045834000
H	2.690395000	-3.298921000	-2.148926000
H	1.721238000	-3.614156000	-3.638455000
C	-0.943234000	-2.362322000	-3.039501000
H	-0.752937000	-2.491831000	-4.116140000
H	-1.622998000	-1.515156000	-2.871766000
H	-1.403944000	-3.273182000	-2.630248000
C	3.852692000	-0.628840000	-1.558121000
H	3.471109000	-1.370400000	-0.840728000
H	4.595985000	-0.000935000	-1.046236000
H	4.303341000	-1.129945000	-2.429061000
C	3.202386000	1.595753000	-3.307632000
H	3.737060000	1.035022000	-4.090062000
H	3.892703000	2.265112000	-2.773567000
H	2.403296000	2.201930000	-3.758890000

6 – B3PW91-D3(BJ)/cc-pVDZ,aug-cc-pVDZ-PP{Pt}

Br	3.776002000	2.631653000	0.320263000
Pt	1.493753000	1.471459000	-0.225071000
C	-0.549876000	0.910362000	2.487015000
H	-1.551008000	0.850781000	2.033297000
H	-0.661878000	1.165184000	3.552904000
P	0.367390000	2.254170000	1.591512000
Br	1.249950000	-3.848112000	1.053603000
Pt	0.359201000	-1.557630000	0.127523000
C	1.387518000	-0.658090000	-3.038322000
H	1.928698000	-1.057147000	-3.911152000
H	0.578791000	-0.001385000	-3.396301000
P	0.597055000	-2.040397000	-2.084469000
P	2.491675000	0.409664000	-2.001167000
P	0.206153000	-0.769463000	2.278572000
B	-0.152741000	0.321593000	-0.502101000

N	-1.447511000	0.653713000	-0.925851000
Si	-5.153003000	1.966811000	-0.999860000
C	-6.265775000	1.438115000	-2.418751000
H	-5.660779000	1.098270000	-3.274081000
H	-6.885794000	2.285799000	-2.753267000
H	-6.939349000	0.618974000	-2.127446000
B	-2.716793000	0.666183000	-0.785979000
N	-4.077138000	0.595268000	-0.557800000
Si	-4.557577000	-0.874328000	0.392933000
C	-6.173727000	2.457828000	0.501733000
H	-6.827436000	1.641359000	0.844551000
H	-6.817088000	3.316998000	0.251827000
H	-5.520724000	2.752154000	1.338108000
C	-3.316271000	-2.223792000	0.015552000
H	-3.406360000	-2.543454000	-1.033995000
H	-3.503963000	-3.101902000	0.654298000
H	-2.266749000	-1.906420000	0.178699000
C	-6.293584000	-1.389886000	-0.112202000
H	-7.043590000	-0.611367000	0.094108000
H	-6.581238000	-2.288623000	0.457373000
H	-6.334596000	-1.640218000	-1.183398000
C	-4.521565000	-0.422920000	2.220019000
H	-3.523587000	-0.057930000	2.507814000
H	-4.750225000	-1.305689000	2.839150000
H	-5.253736000	0.362320000	2.460730000
C	-4.051683000	3.390158000	-1.536268000
H	-3.439844000	3.762561000	-0.700532000
H	-4.671184000	4.224817000	-1.902199000
H	-3.370089000	3.089671000	-2.347665000
C	1.353678000	3.052923000	2.909059000
H	0.711831000	3.392218000	3.735956000
H	1.894942000	3.897832000	2.461207000
H	2.107210000	2.342594000	3.273308000
C	-0.960553000	3.464753000	1.234803000

H	-1.604822000	3.023027000	0.462299000
H	-0.498452000	4.376209000	0.829566000
H	-1.545535000	3.708890000	2.134395000
C	1.866140000	-0.620337000	3.029622000
H	1.817461000	-0.236564000	4.059846000
H	2.474761000	0.042074000	2.395229000
H	2.313032000	-1.624908000	3.012852000
C	-0.748792000	-1.798164000	3.453282000
H	-0.329842000	-2.813667000	3.395084000
H	-1.797650000	-1.834962000	3.127448000
H	-0.686892000	-1.410771000	4.481320000
C	1.615733000	-3.500305000	-2.507339000
H	1.175213000	-4.373595000	-2.006300000
H	2.624275000	-3.365571000	-2.095369000
H	1.662237000	-3.653391000	-3.596011000
C	-0.963487000	-2.314902000	-3.005133000
H	-0.782651000	-2.438369000	-4.083421000
H	-1.618246000	-1.451689000	-2.822097000
H	-1.444006000	-3.217768000	-2.602515000
C	3.797460000	-0.725879000	-1.409840000
H	3.344516000	-1.436014000	-0.701220000
H	4.529798000	-0.112192000	-0.865720000
H	4.281668000	-1.260149000	-2.241243000
C	3.325527000	1.473120000	-3.234626000
H	3.876659000	0.878518000	-3.978655000
H	4.012983000	2.125740000	-2.676580000
H	2.574306000	2.101087000	-3.734165000

mod-1 – ωB97XD/cc-pVDZ,aug-cc-pVDZ-PP{Pt}

Br	3.713505000	-1.981474000	-0.454883000
Pt	1.666761000	-0.348602000	-0.194079000
C	0.416632000	0.273205000	3.008619000
H	0.079309000	1.318087000	2.915012000
H	0.611245000	0.066507000	4.073176000

P	1.990393000	0.122073000	2.032453000
Br	-3.627859000	-2.116743000	0.502249000
Pt	-1.649090000	-0.410003000	0.194998000
C	-0.422634000	0.174931000	-3.023627000
H	-0.607774000	-0.067103000	-4.082415000
H	-0.128250000	1.234790000	-2.957297000
P	-1.989921000	-0.014034000	-2.043695000
P	1.024865000	-0.788438000	-2.376615000
P	-0.989988000	-0.765275000	2.388038000
B	-0.014809000	0.789480000	-0.014618000
N	-0.042332000	2.203799000	-0.030913000
Si	1.418104000	5.791288000	-0.182950000
B	-0.057400000	3.469265000	-0.038964000
N	-0.099410000	4.861657000	-0.044637000
Si	-1.686082000	5.663998000	0.120584000
C	2.981181000	-1.076809000	2.997060000
H	3.102209000	-0.744131000	4.038691000
H	3.957913000	-1.181546000	2.504294000
H	2.492227000	-2.059513000	2.967008000
C	2.813395000	1.725985000	2.362381000
H	2.243689000	2.515008000	1.851795000
H	3.823608000	1.687355000	1.930461000
H	2.875773000	1.937635000	3.440186000
C	-0.419944000	-2.487604000	2.627810000
H	-0.071916000	-2.661768000	3.657141000
H	0.385832000	-2.696308000	1.908321000
H	-1.268622000	-3.146288000	2.393595000
C	-2.264308000	-0.522682000	3.678307000
H	-3.133238000	-1.133958000	3.394401000
H	-2.569627000	0.533164000	3.687915000
H	-1.895489000	-0.816832000	4.672045000
C	-2.928912000	-1.279250000	-2.974653000
H	-3.902432000	-1.408244000	-2.481354000
H	-2.401567000	-2.240484000	-2.914955000

H	-3.059808000	-0.982082000	-4.025783000
C	-2.878101000	1.544372000	-2.418941000
H	-2.949342000	1.721472000	-3.502413000
H	-2.341593000	2.371404000	-1.933163000
H	-3.885900000	1.476830000	-1.984921000
C	0.527765000	-2.538685000	-2.572604000
H	-0.269441000	-2.763234000	-1.848442000
H	1.403192000	-3.154774000	-2.321502000
H	0.188847000	-2.753349000	-3.597302000
C	2.289479000	-0.526366000	-3.672706000
H	1.933672000	-0.859620000	-4.658821000
H	3.182189000	-1.094876000	-3.374073000
H	2.551905000	0.540386000	-3.708390000
H	-1.463319000	7.104190000	-0.205417000
H	-2.197686000	5.529882000	1.515207000
H	-2.650791000	5.051140000	-0.836745000
H	2.541377000	4.813336000	-0.149579000
H	1.511522000	6.745638000	0.961937000
H	1.423706000	6.562653000	-1.460843000

mod-2 – ωB97XD/cc-pVDZ,aug-cc-pVDZ-PP{Pt}

Br	-3.985432000	-1.697227000	-0.000087000
Pt	-1.744056000	-0.381904000	0.000459000
C	-0.000557000	0.066863000	-3.089829000
H	0.080145000	1.155650000	-2.943941000
H	-0.016729000	-0.147745000	-4.167155000
P	-1.594083000	-0.447730000	-2.286823000
Br	3.676701000	-2.273758000	-0.002396000
Pt	1.658300000	-0.636592000	-0.000375000
C	0.000876000	0.061890000	3.090817000
H	-0.014758000	-0.154453000	4.167803000
H	0.081018000	1.150944000	2.946620000
P	1.501722000	-0.679750000	2.287005000
P	-1.592718000	-0.452179000	2.287606000

P	1.500230000	-0.676804000	-2.287778000
B	0.042176000	0.627283000	0.001031000
N	0.147487000	2.029610000	0.002310000
Si	-1.095371000	5.706634000	-0.004753000
B	0.237990000	3.294566000	0.003250000
N	0.366890000	4.677389000	0.004380000
Si	2.010924000	5.383314000	0.005310000
H	-1.799695000	-1.736163000	-2.843566000
H	-2.526171000	0.291645000	-3.060296000
H	1.516131000	-1.980285000	-2.847322000
H	2.530691000	-0.079718000	-3.059185000
H	1.518840000	-1.983925000	2.844897000
H	2.532188000	-0.082918000	3.058600000
H	-1.797216000	-1.741855000	2.841937000
H	-2.524910000	0.285064000	3.062955000
H	1.821236000	6.861897000	0.084946000
H	2.723530000	5.018437000	-1.252260000
H	2.770899000	4.887689000	1.187650000
H	-2.273544000	4.796661000	-0.040145000
H	-1.065189000	6.589902000	-1.207621000
H	-1.110320000	6.553316000	1.224259000

mod-3 – ωB97XD/cc-pVDZ,aug-cc-pVDZ-PP{Pt}

Br	3.662609000	0.511334000	-1.589434000
Pt	1.654978000	0.209143000	0.085969000
C	0.446000000	-3.013122000	0.690332000
H	0.134493000	-2.930991000	1.744134000
H	0.643741000	-4.074089000	0.468555000
P	2.007146000	-2.022451000	0.510066000
Br	-3.662841000	-0.511542000	-1.588624000
Pt	-1.655082000	-0.209151000	0.086472000
C	-0.446062000	3.013127000	0.689786000
H	-0.643823000	4.074070000	0.467910000
H	-0.134135000	2.931168000	1.743483000

P	-2.007281000	2.022477000	0.510353000
P	0.989722000	2.392717000	-0.307132000
P	-0.990161000	-2.392954000	-0.306180000
B	0.000027000	0.000085000	1.263104000
N	0.000721000	0.000216000	2.674289000
B	0.001578000	0.000607000	3.941555000
N	0.003131000	0.001277000	5.323576000
C	2.969740000	-2.959615000	-0.732980000
H	3.103920000	-4.006690000	-0.423090000
H	3.941506000	-2.460797000	-0.853745000
H	2.455435000	-2.913835000	-1.701959000
C	2.878986000	-2.375822000	2.082678000
H	2.326585000	-1.887900000	2.897820000
H	3.883537000	-1.933132000	2.023639000
H	2.957880000	-3.457078000	2.269961000
C	-0.463728000	-2.613387000	-2.045018000
H	-0.110637000	-3.637812000	-2.237071000
H	0.329007000	-1.884336000	-2.269436000
H	-1.332021000	-2.381787000	-2.678599000
C	-2.249319000	-3.694530000	-0.044199000
H	-3.134890000	-3.411331000	-0.631457000
H	-2.528376000	-3.715912000	1.018702000
H	-1.881076000	-4.682949000	-0.356537000
C	-2.970341000	2.959264000	-0.732596000
H	-3.942483000	2.460901000	-0.852218000
H	-2.456855000	2.912437000	-1.701959000
H	-3.103713000	4.006638000	-0.423380000
C	-2.878574000	2.376277000	2.083167000
H	-2.957469000	3.457583000	2.270139000
H	-2.325856000	1.888663000	2.898278000
H	-3.883127000	1.933517000	2.024657000
C	0.462523000	2.612647000	-2.045797000
H	-0.330236000	1.883459000	-2.269685000
H	1.330567000	2.380975000	-2.679695000

H	0.109243000	3.636983000	-2.237972000
C	2.248814000	3.694517000	-0.045987000
H	1.880294000	4.682833000	-0.358323000
H	3.134174000	3.411332000	-0.633572000
H	2.528349000	3.716112000	1.016786000
H	-0.836485000	-0.133617000	5.868706000
H	0.844137000	0.136639000	5.866309000

mod-4 – ωB97XD/cc-pVDZ,aug-cc-pVDZ-PP{Pt}

Br	-3.829875000	-1.467592000	0.004623000
Pt	-1.703601000	0.026905000	-0.000134000
C	0.000036000	0.598183000	-3.091237000
H	0.000133000	1.689947000	-2.945476000
H	0.000050000	0.381781000	-4.168314000
P	-1.550625000	-0.031813000	-2.287054000
Br	3.829903000	-1.467610000	0.004514000
Pt	1.703553000	0.026784000	-0.000041000
C	-0.000068000	0.617533000	3.087413000
H	-0.000086000	0.407873000	4.165825000
H	-0.000067000	1.708365000	2.934831000
P	1.550522000	-0.017656000	2.287190000
P	-1.550615000	-0.017656000	2.287119000
P	1.550528000	-0.032125000	-2.286963000
B	0.000002000	1.174310000	-0.003665000
N	0.000133000	2.577622000	-0.008033000
B	0.000277000	3.846685000	-0.011923000
N	0.000427000	5.225624000	-0.016152000
H	-1.661391000	-1.332385000	-2.842574000
H	-2.535277000	0.635760000	-3.060710000
H	1.660954000	-1.332816000	-2.842293000
H	2.535373000	0.635077000	-3.060690000
H	1.661073000	-1.314789000	2.850721000
H	2.535250000	0.654524000	3.056757000
H	-1.661134000	-1.314837000	2.850528000

H	-2.535399000	0.654435000	3.056689000
H	0.852208000	5.768980000	-0.017684000
H	-0.851253000	5.769138000	-0.017883000

References

[1] C. Brunecker, J. H. Mussig, M. Arrowsmith, F. Fantuzzi, A. Stoy, J. Bohnke, A. Hofmann, R. Bertermann, B. Engels, H. Braunschweig, *Chem. Eur. J.* **2020**, *26*, 8518–8523.

[2] W. Haubold, U. Kraatz, *Z. anorg. allg. Chem.* **1976**, *421*, 105–110.

[3] P. Geymayer, E. G. Rochow, *Monatsh. Chem.* **1966**, *97*, 429–436.

[4] R. H. Neilson, R. L. Wells, *Synth. React. Inorg. Metal-Org. Chem.* **1973**, *3*, 283–289.

[5] H. Braunschweig, K. Radacki, A. Schneider, *Science* **2010,** *328*, 345–347.

[6] G. Sheldrick, *Acta Cryst.* **2015**, *A71*, 3–8.

[7] G. Sheldrick, *Acta Cryst.* **2008**, *A64*, 112–122.

[8] A. L. Spek, *Acta Cryst*. **2015**, *C71*, 9–18.

[9] Y. Zhao, D. G. Truhlar, *Theor. Chem. Acc.* **2008**, *120*, 215–241.

[10] a) C. Adamo, V. Barone, *J. Chem. Phys.* **1999**, *110*, 6158–6170; b) M. Ernzerhof, G. E. Scuseria, *J. Chem. Phys.* **1999**, *110*, 5029–5036.

[11] J.-D. Chai, M. Head-Gordon, *Phys. Chem. Chem. Phys.* **2008**, *10*, 6615–6620.

[12] a) S. H. Vosko, L. Wilk, M. Nusair, *Can. J. Phys.* **1980**, *58*, 1200–1211; b) C. Lee, W. Yang, R. G. Parr, *Phys. Rev. B* **1988**, *37*, 785–789; c) A. D. Becke, *J. Chem. Phys.* **1993**, *98*, 5648–5652; d) P. J. Stephens, F. J. Devlin, C. F. Chabalowski, M. J. Frisch, *J. Phys. Chem.* **1994**, *98*, 11623–11627.

[13] a) J. P. Perdew, in *Electron. Struct. Solids' 91* (Eds.: P. Ziesche, H. Eschrig), Akademie Verlag, Berlin, **1991**, p. 11; b) J. P. Perdew, K. Burke, Y. Wang, *Phys. Rev. B* **1996**, *54*, 16533–16539.

[14] a) T. H. Dunning, *J. Chem. Phys.* **1989**, *90*, 1007–1023; b) D. E. Woon, T. H. Dunning, *J. Chem. Phys.* **1993**, *98*, 1358–1371; c) A. K. Wilson, D. E. Woon, K. A. Peterson, T. H. Dunning, *J. Chem. Phys.* **1999**, *110*, 7667–7676.

[15] D. Figgen, K. A. Peterson, M. Dolg, H. Stoll, *J. Chem. Phys.* **2009**, *130*, 164108.

[16] S. Grimme, J. Antony, S. Ehrlich, H. Krieg, *J. Chem. Phys.* **2010**, *132*, 154104.

[17] S. Grimme, S. Ehrlich, L. Goerigk, *J. Comput. Chem.* **2011**, *32*, 1456–1465.

[18] a) I. Mayer, *Chem. Phys. Lett.* **1983**, *97*, 270–274; b) I. Mayer, *Int. J. Quantum Chem.* **1984**, *26*, 151–154.

[19] G. Knizia, *J. Chem. Theory Comput.* **2013**, *9*, 4834–4843.

[20] M. J. Frisch, G. W. Trucks, H. B. Schlegel, G. E. Scuseria, M. A. Robb, J. R. Cheeseman, G. Scalmani, V. Barone, B. Mennucci, G. A. Petersson, H. Nakatsuji, M.

Caricato, X. Li, H. P. Hratchian, A. F. Izmaylov, J. Bloino, G. Zheng, J. L. Sonnenberg, M. Hada, M. Ehara, K. Toyota, R. Fukuda, J. Hasegawa, M. Ishida, T. Nakajima, Y. Honda, O. Kitao, H. Nakai, T. Vreven, J. A. Montgomery Jr., J. E. Peralta, F. Ogliaro, M. Bearpark, J. J. Heyd, E. Brothers, K. N. Kudin, V. N. Staroverov, R. Kobayashi, J. Normand, K. Raghavachari, A. Rendell, J. C. Burant, S. S. Iyengar, J. Tomasi, M. Cossi, N. Rega, J. M. Millam, M. Klene, J. E. Knox, J. B. Cross, V. Bakken, C. Adamo, J. Jaramillo, R. Gomperts, R. E. Stratmann, O. Yazyev, A. J. Austin, R. Cammi, C. Pomelli, J. W. Ochterski, R. L. Martin, K. Morokuma, V. G. Zakrzewski, G. A. Voth, P. Salvador, J. J. Dannenberg, S. Dapprich, A. D. Daniels, Ö. Farkas, J. B. Foresman, J. V Ortiz, J. Cioslowski, D. J. Fox, *Gaussian 16, Revision B.01*, Gaussian, Inc., Wallingford CT, **2016**.
